# Molecular characterization of three novel perforins in common carp (*Cyprinus carpio* L.) and their expression patterns during larvae ontogeny and in response to immune challenges

**DOI:** 10.1186/s12917-018-1613-y

**Published:** 2018-10-03

**Authors:** Ting Li, Lei Wang, Yonghuan Zhang, Xinyi Guo, Xinze Chen, Fumiao Zhang, Guiwen Yang, Wujun Wen, Hua Li

**Affiliations:** 1 Shandong Provincial Key Laboratory of Animal Resistance Biology, College of Life Sciences, Shandong Normal University, Jinan, 250014 China; 2 Key Laboratory for Sustainable Development of Marine Fisheries, Ministry of Agriculture, Yellow Sea Fisheries Research Institute, Chinese Academy of Fishery Sciences, Qingdao, 266071 China; 3 Laboratory for Marine Fisheries Science and Food Production Processes, Qingdao National Laboratory for Marine Science and Technology, Qingdao, 266237 China; 4 National Life Science and Technology Training Base, Nanjing Agricultural University, Nanjing, 210000 China

**Keywords:** Common carp (*Cyprinus carpio* L.), Perforin, Evolutionary relationship, Expression pattern

## Abstract

**Background:**

In the host immune system, perforin is a cytotoxic effector molecule that eliminate virus-infected and malignant cells. Moreover, some recent studies also imply the involvement of perforin in antibacterial immunity. Common carp (*Cyprinus carpio* L.), one of the most economically important fish species in China, has a high susceptibility to viruses and bacteria. Thus far, in common carp, no data are available regarding the identification and immunologic function of the perforin.

**Results:**

In the present study, the cDNA and genomic DNA sequences of three perforin isoform genes were cloned and characterized in common carp, named *Cc*PRF1, *Cc*PRF2 and *Cc*PRF3. Amino acid sequences of the three *Cc*PRFs were quite different, with identities ranged from 37.3 to 39.5%. Phylogenetic analysis showed that three *Cc*PRFs, each in a separate sub-branch, possessed closer evolutionary relationship with other teleost perforins, especially with cyprinid fishes, than higher vertebrates. Expression analysis revealed that each *Cc*PRF gene was differentially expressed in all of the nine tested tissues. During larvae ontogeny, each *Cc*PRF displayed a distinct expression pattern, while with a common expression peak at 22 days post hatching (dph). Moreover, in vivo or in vitro, after stimulation with polyI:C, LPS and *Aeromonas hydrophila*, each *Cc*PRF was induced significantly, with differential expression dynamics.

**Conclusions:**

Our findings suggest that perforin might play significant roles in larval immune system and in the immune defense of common carp against viral and bacterial pathogens. Meantime, the differential expression dynamics seem to imply possible different cellular locations or functional differences across various *Cc*PRF isoforms.

**Electronic supplementary material:**

The online version of this article (10.1186/s12917-018-1613-y) contains supplementary material, which is available to authorized users.

## Background

In mammals, natural killer (NK) cells and CD8^+^ cytotoxic T lymphocytes (CD8^+^ CTLs) play important roles in the immune-cytotoxic system against virus-infected and tumor cells. In this process, it has been confirmed that perforin plays a crucial role [1]. Moreover, some recent studies pointed also to the role of perforin in resisting bacterial pathogens [2, 3]. Until now, in addition to these two cytotoxic cells, some studies reported the expression of perforin in other types of cell, including NKT cells [4], effector-like γδ T cells [5] and some CD4^+^ T lymphocytes [6].

Perforin is a 60–70 kDa pore-forming glycoprotein, which belongs to the membrane-attack-complex/perforin (MACPF) family [7, 8]. Mature perforin molecule has three domains: an N-terminal MACPF domain, an intermediate epidermal growth factor (EGF)-like domain and a C-terminal calcium-binding (CalB) domain. Pore formation of perforin depends on these two characteristic domains, in which MACPF is related to pore-forming [9, 10] and the CalB motif is related to calcium-dependent membrane binding to target cells [11]. Under normal circumstances, perforin is stored in cytoplasmic secretory granules of NK cells and CTLs. After these cytotoxic cells are activated, monomer perforin polymerizes to form a channel of 5–20 nm in diameter in target cell membranes, leading to the disruption of cell membrane and subsequently the death of target cells [12]. Additionally, the pores formed by perforin allow granzymes (Gzms), serine proteases stored in cytoplasmic granules of NK/CTL cells with perforin, into the cytosol of target cells [13], which will induce the activation of proapoptotic pathways and DNA degradation.

Perforin was first characterized as a lytic pore-forming protein isolated from CTLs in 1985 [14]. In humans and mice, perforin is a single-copy gene, and presently its immunological function has been well studied. In teleosts, until now, the perforin genes have been characterized in 6 species, including Japanese flounder (*Paralichtys olivaceus*) [15, 16], channel catfish (*Ictalurus punctatus*) [17], rainbow trout (*Oncorhynchus mykiss*) [18], ginbuna crucian carp (*Carassius auratus langsdorfii*) [19], rock bream (*Oplegnathus fasciatus*) [20, 21] and zebrafish (*Danio rerio*) [22]. Moreover, different with humans and mice, there are more than one perforin genes in fish genomes [19, 22], although only one isoform was reported in some teleosts to date [16–18, 20].

Common carp (*Cyprinus carpio* L.) is one of the most important aquaculture fish species in China and has a high susceptibility to viruses and bacteria. In common carp, although there were data suggesting the existence of a perforin/Gzm pathway like mammals [23], no data are currently available regarding the identification and immune function of the perforin. In the present study, three perforin isoform genes were cloned and characterized from common carp, named *Cc*PRF1, *Cc*PRF2 and *Cc*PRF3 according to their amino acid identities with crucian carp perforins. We studied their evolution and analysed their constitutive expression patterns in healthy carps and during larvae ontogeny. Moreover, gene expression patterns of the three *Cc*PRFs upon polyinosinic-polycytidylic acid (polyI:C) and *Aeromonas hydrophila* (*A. hydrophila*) stimulation were determined in vivo*.* Meanwhile, we isolated leukocytes from the head kidney and peripheral blood of common carp, and analysed polyI:C and lipopolysaccharides (LPS)-induced expression of *Cc*PRFs and CD8α gene in vitro.

## Methods

### Fish rearing and sample collection

Healthy common carps, with a body weight of 80 ± 5 g, were obtained from the Fresh Water Fishery Research Institute of Shandong Province, China. Before the start of experiments, fish were acclimated to the laboratory environment in a recirculating freshwater system at 20 °C for 2 weeks and fed once daily with commercial carp pellets. Four healthy fish were euthanatized by anesthesia in 100 mg/L MS222 (Sigma, USA), and the tissue samples, including those of the liver, spleen, head kidney, foregut, hindgut, gill, skin, brain and muscle, were isolated for RNA extraction.

To investigate gene expression patterns of three *Cc*PRFs during embryonic and early larval stages, four pairs of parent fish were selected for artificial propagation, which was performed based on a previous study [24]. Fertilized eggs were incubated in a water tank at 28–30 °C with enough oxygen. At 2 days post hatching (dph), the larvae started to be fed with aseptic soymilk. During 9 dph-22 dph, the juvenile fish were fed with fine-grained fish meal. At various developmental stages with blastula stage (1 days post fertilization, 1 dpf), optic primordium appearance (2 dpf), 1 dph, 2 dph, 4 dph, 8 dph, 14 dph and 22 dph, five embryos or larvae were sampled randomly for RNA extraction.

### RNA extraction and cDNA preparation

Total RNA was extracted from the tissue, embryo and larva samples using the RNAsimple Total RNA Kit (Tiangen, China). The concentration and quality of total RNA was measured using a spectrophotometer. The RNA template was subjected to reverse transcription into first-strand cDNA using the FastQuent RT Kit (with gDNase) (Tiangen, China).

### cDNA and genomic DNA cloning of *Cc*PRF1, *Cc*PRF2 and *Cc*PRF3 genes

To clone the cDNA sequences of perforin from common carp, three pairs of primers, PRF1-F/PRF1-R, PRF2-F/PRF2-R and PRF3-F/PRF3-R, were designed based on the conserved regions of published perforin cDNA sequences from other fish species. The first-strand cDNA synthesized by spleen-derived RNA served as a template to amplify the corresponding cDNA sequences of *Cc*PRF1, *Cc*PRF2 and *Cc*PRF3, and a 405-bp cDNA fragment of CcPRF1, a 611-bp of CcPRF2 and a 1367-bp of CcPRF3 were obtained. PCR was performed using the following steps: denaturation at 95 °C for 5 min, followed by 35 cycles of 95 °C for 30 s, 60 °C for 30 s, and 72 °C for 2 min, with a final extension step of 72 °C for 10 min. Next, the full-length cDNA sequences of *Cc*PRF1, *Cc*PRF2 and *Cc*PRF3 were obtained by the rapid amplification of the cDNA ends (RACE) method with a 5’-Full RACE and 3’-Full RACE Core Set Kit (TaKaRa, Japan). The procedure was carried out as described in the user manual, and two rounds of PCR were performed to amplify the 5′ and 3′ flanking regions. The primers used for cDNA cloning are shown in Additional file 1: Table S1.

To obtain the DNA sequences of three *Cc*PRF genes, genomic DNA was purified from the spleen of common carp with the Genomic DNA Kit (Tiangen, China). Based on *Cc*PRF1, *Cc*PRF2 and *Cc*PRF3 cDNA sequences, three, four and three primer pairs were designed respectively, and then their PCR products were analysed and sequenced. The primers used for DNA cloning are shown in Additional file 2: Table S2.

All PCR products were analysed by 1% agarose electrophoresis, and the anticipated fragments were purified using the PCR purification kit (Tiangen, China). These fragments were then ligated into the pMD18-T vector (TaKaRa, Japan), transformed into competent *Escherichia coli* cells DH-5α and sequenced by BGI China.

### PolyI:C and *A. hydrophila* stimulation in vivo

The protocols for the immune challenges were carried out as described in previous studies. PolyI:C (Sigma, USA) was suspended in sterile phosphate-buffered saline (PBS) at a final density of 1.6 mg/ml, and twenty-one fish (body mass 80 ± 5 g) were injected intraperitoneally with polyI:C at 500 μl per fish [25–27]. *A. hydrophila* was incubated in Luria-Bertani medium at 28 °C overnight under continuous shaking. *A. hydrophila* was inactivated in 0.5% formaldehyde at 4 °C overnight and was subsequently suspended in sterile PBS at a final density of 2.0 × 108 CFU/ml. Twenty-one carps whose body weight was 80 ± 5 g were injected intraperitoneally with 500 μl of inactivated *A. hydrophila* [28–30].

For the polyI:C-challenged carps, at 3 h, 6 h, 12 h, 24 h, 48 h, 72 h and 120 h post injection (hpi), three carp individuals were euthanized, and the liver, spleen, head kidney, foregut, hindgut and skin were sampled and kept in liquid nitrogen for total RNA extraction. For *A. hydrophila*-challenged carps, at 3, 6, 12, 24, 72 and 168 hpi, three fish were dissected, and the same tissues as those of polyI:C-stimulated group were selected for analysis. In addition, three un-stimulated fish served as control group.

### Isolation and stimulation of peripheral blood leukocytes (PBLs) and head kidney leukocytes (HKLs)

The method for isolating common carp PBLs was described in previous reports [31]. Heparinized blood samples were taken from the caudal blood vessel and diluted with an equal volume of complete media (incomplete L-15 medium (Gibco, USA) containing 10% fetal calf serum (FCS), 100 U/ml penicillin and 100 μg/ml streptomycin). Then, the resulting diluted blood was placed onto 65% percoll density cushions (Sigma, USA) and centrifuged at 2500 rpm for 30 min at normal temperature. The layer of PBLs was collected and washed three times with PBS. The method for isolating common carp HKLs was performed based on previously described protocols [32]. Healthy common carp, with a body weight of 80 g, was obtained from the Fresh Water Fishery Research Institute of Shandong Province and raised in a recirculating freshwater system. The tissue head kidney was collected from freshly killed carp under sterile conditions, pressed with a plunger through a 100-μm sterile nylon mesh and rinsed with incomplete Leibovitz’s L-15 medium (Gibco, USA). Cell isolation was performed using a 34/51% non-continuous Percoll gradient (Sigma, USA). After 30 min of centrifugation at 1500 rpm, the cell layer present in interphase was collected and then washed three times with PBS. Finally, the cells isolated from heparinized blood and head kidney were individually resuspended in the complete media and cultured in 24-well plates at 25 °C.

After incubation overnight, drug treatment of polyI:C and LPS (10 μg/ml) (Sigma, USA) was performed in these two groups. At 3 h, 6 h, 12 h and 24 h post stimulation, the cells were harvested for RNA extraction. Additionally, the un-stimulated cells served as control group.

### Real-time PCR

Gene expression analyses of *Cc*PRF1, *Cc*PRF2, *Cc*PRF3, and CD8α were performed on a LightCycler® 96 Real-time PCR System (Roche, Switzerland) using *TransStart*® Tip Green qPCR SuperMix (Transgen, China). The program was as follows: 94 °C for 30 s, followed by 40 cycles of 94 °C for 5 s, 58 °C for 15 s and 72 °C for 10 s. Finally, melt curve analysis was carried out to verify the presence of one single PCR product at the end of the assay. Each sample was analysed in triplicate, and the 40S ribosomal protein S11 gene served as a housekeeping gene to normalize the mRNA expression. The relative gene expression was determined using the 2^-∆∆CT^ method [33], and the primers used in Real-time PCR are shown in Additional file 3: Table S3.

### Bioinformatics and statistical analyses

The putative open reading frames (ORFs) and protein prediction were analysed with EditSeq within DNASTAR. The protein domains were predicted using the simple modular architecture research tool (SMART) (http://smart.embl-heidelberg.de/). Phylogenetic trees were generated with MEGA 6.0 using the neighbor-joining (NJ) method, in which the Jones-Taylor-Thornton (JTT) model was used as an amino acid substitution model. The protein tertiary structures were predicted by SWISS-MODEL (https://swissmodel.expasy.org/). GeneBank accession numbers used in phylogenetic and homology analyses are shown in Additional file 4: Table S4 and Additional file 5: Table S5.

The differences in relative gene expression between the stimulated group and control group (0 h) were conducted using two-way analysis of variance (ANOVA) in Graphpad Prism 5.0 and were considered significant when *p* < 0.05.

## Results

### Characterization of three *Cc*PRF cDNA sequences

**Fig. 1:**
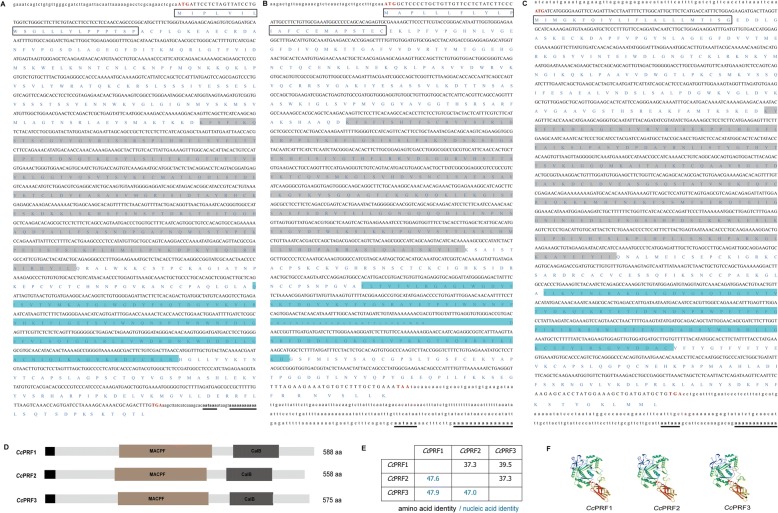
Molecular characteristic of the three *Cc*PRF genes. **a**-**c** cDNA and deduced amino acid sequences. *Cc*PRF1, *Cc*PRF2 and *Cc*PRF3 are shown in **a**, **b** and **c**, respectively. The translation start codon ATG and termination codons TGA or TAA are shown in red. The signal peptides are boxed, and the MACPF and CalB domains are highlighted in light gray and blue, respectively. In addition, the polyadenylation signal (aataaa) and poly (**a**) tail are in bold and underlined. **d** Domain structures of three *Cc*PRF proteins. The signal peptide (black), MACPF (brown) and CalB (darkgray) are depicted in different colors, and the numbers refer to the length of the amino acid sequences. **e** Amino acid (black) and nucleic acid (green) identities among the three *Cc*PRFs. **f** 3-Dimensional protein structures were predicted using the SWISS-MODEL server, with selection of the model based on the best C-score

The full-length cDNA sequences of *Cc*PRF1, *Cc*PRF2 and *Cc*PRF3 were 1869 bp, 1971 bp and 1966 bp (Fig. 1a, b,  c), and their GenBank accession numbers were MH271082, MH271083 and MH271084, respectively. *Cc*PRF1 comprised a 60-bp 5′-untranslated region (UTR), an ORF of 1767 bp encoding 588 amino acids and a 42-bp 3’-UTR. *Cc*PRF2 comprised a 40-bp 5’-UTR, an ORF of 1677 bp encoding 558 amino acids and a 254-bp 3’-UTR. *Cc*PRF3 was composed of a 66-bp 5’-UTR, an ORF of 1728 bp encoding 575 amino acids and a 172-bp 3’-UTR. Protein domain prediction showed that each *Cc*PRF contained a signal peptide domain, a MACPF domain and a CalB domain (Fig. 1d). The locations of the MACPF domain for *Cc*PRF1, *Cc*PRF2 and *Cc*PRF3 were residues 163–365, 154–354 and 160–358, respectively, and the CalB domains were located at residues 412–511, 398–497 and 405–499, respectively.

### Genomic structures of three *Cc*PRFs

**Fig. 2:**
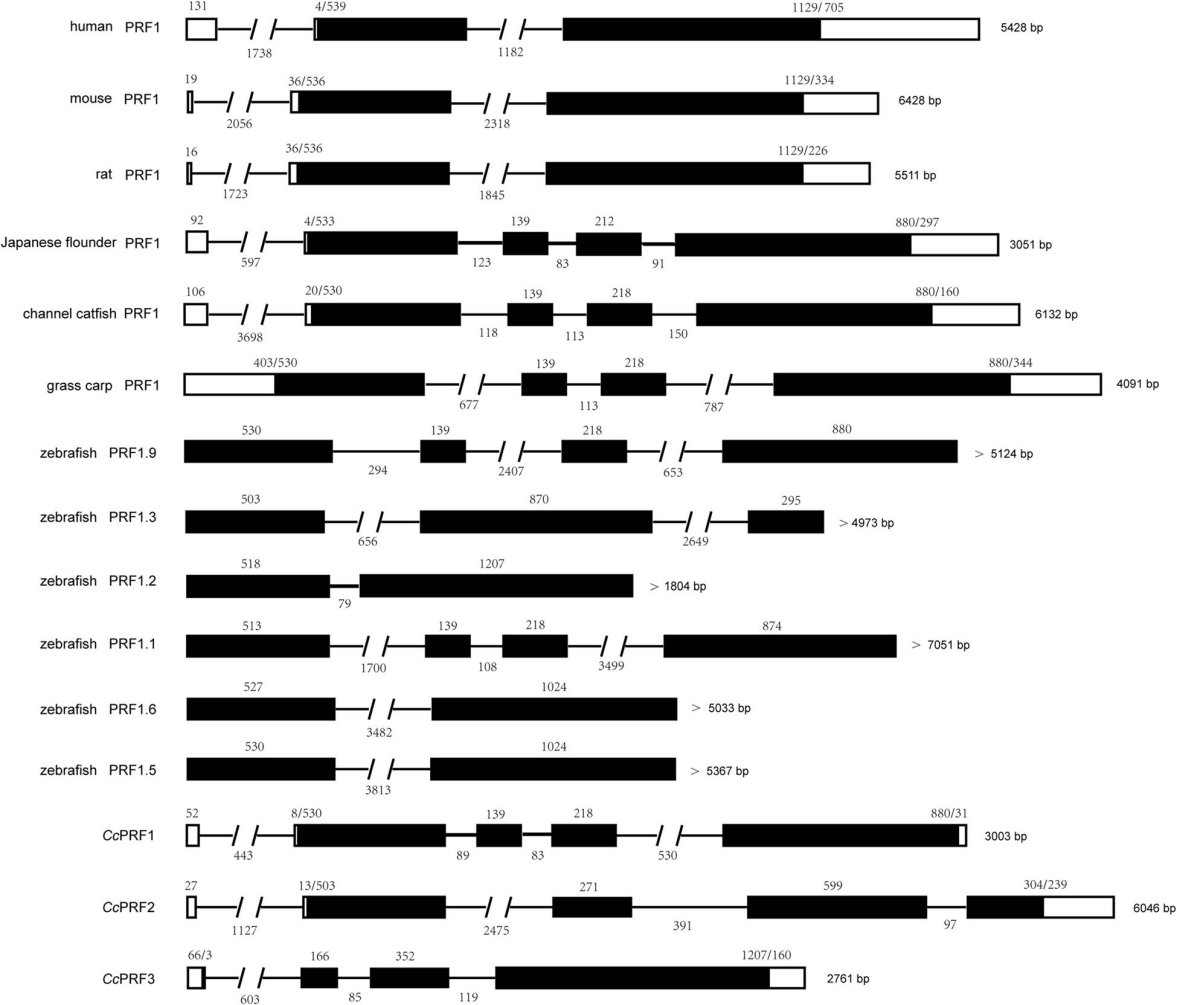
Schematic diagrams of exon-intron arrangement of various vertebrate perforin genes. Exons are shown by boxes, and introns are shown by straight or interrupted lines. The filled boxes show the coding region, while the empty ones show the untranslated region. The number of nucleotides in each exon and intron is shown above or below the corresponding element. The sequences selected are human (gene ID 5551), mouse (gene ID 18646), rat (gene ID 50669), Japanese flounder (refer to [16]), channel catfish (gene ID 108280710), grass carp (GenBank EF635861), zebrafish (gene ID: 569443, 559,849, 103,909,237, 559,384, 100,000,903, 795,573)

*Cc*PRF1 and *Cc*PRF2 genes contained five exons and four introns, which were 3003 bp and 6046 bp, respectively. *Cc*PRF3 DNA was 2761 bp, comprising four exons and three introns. The sizes of exons and introns and their position in DNA were indicated in Fig. 2, and the exon-intron splice junctions followed the AG/GT rule (Table 1). In addition, the specific exon and intron sequences of *Cc*PRF1, *Cc*PRF2 and *Cc*PRF3 are listed in Additional file 6; Additional file 7 and Additional file 8, respectively.

**Table 1:** Intron-exon junctions and flanking sequences of the *Cc*PRF1, *Cc*PRF2 and *Cc*PRF3

Exon no.	Position in DNA	Exon size (bp)	Splice donor	Splice acceptor	Intron size (bp)
*Cc*PRF1–1	1–52	52	**gt**aagtgaaa	atctcagc**ag**	443
2	496–1033	538	**gt**aagactta	ctgtcttc**ag**	89
3	1123–1261	139	**gt**aagccaaa	aatctgtt**ag**	83
4	1345–1562	218	**gt**ttgtgctt	tttctaca**ag**	530
5	2093–3003	911			
*Cc*PRF2–1	1–27	27	**gt**aaatatca	tctctttc**ag**	1127
2	1155–1670	516	**gt**aagttctg	ctttcatc**ag**	2475
3	4146–4416	271	**gt**gagtgggc	gggcgtga**ag**	391
4	4808–5406	599	**gt**aagtggtt	cattttac**ag**	97
5	5504–6046	543			
*C*cPRF3–1	1–69	69	**gt**aagaaact	ttcactac**ag**	603
2	673–838	166	**gt**tcttatgt	gagcgaaa**ag**	85
3	924–1275	352	**gt**tatttttc	ttctttat**ag**	119
4	1395–2761	1367			

Bold text indicates the invariant nucleotides of the exon-intron boundaries

### Phylogenetic and homology analyses

**Fig. 3:**
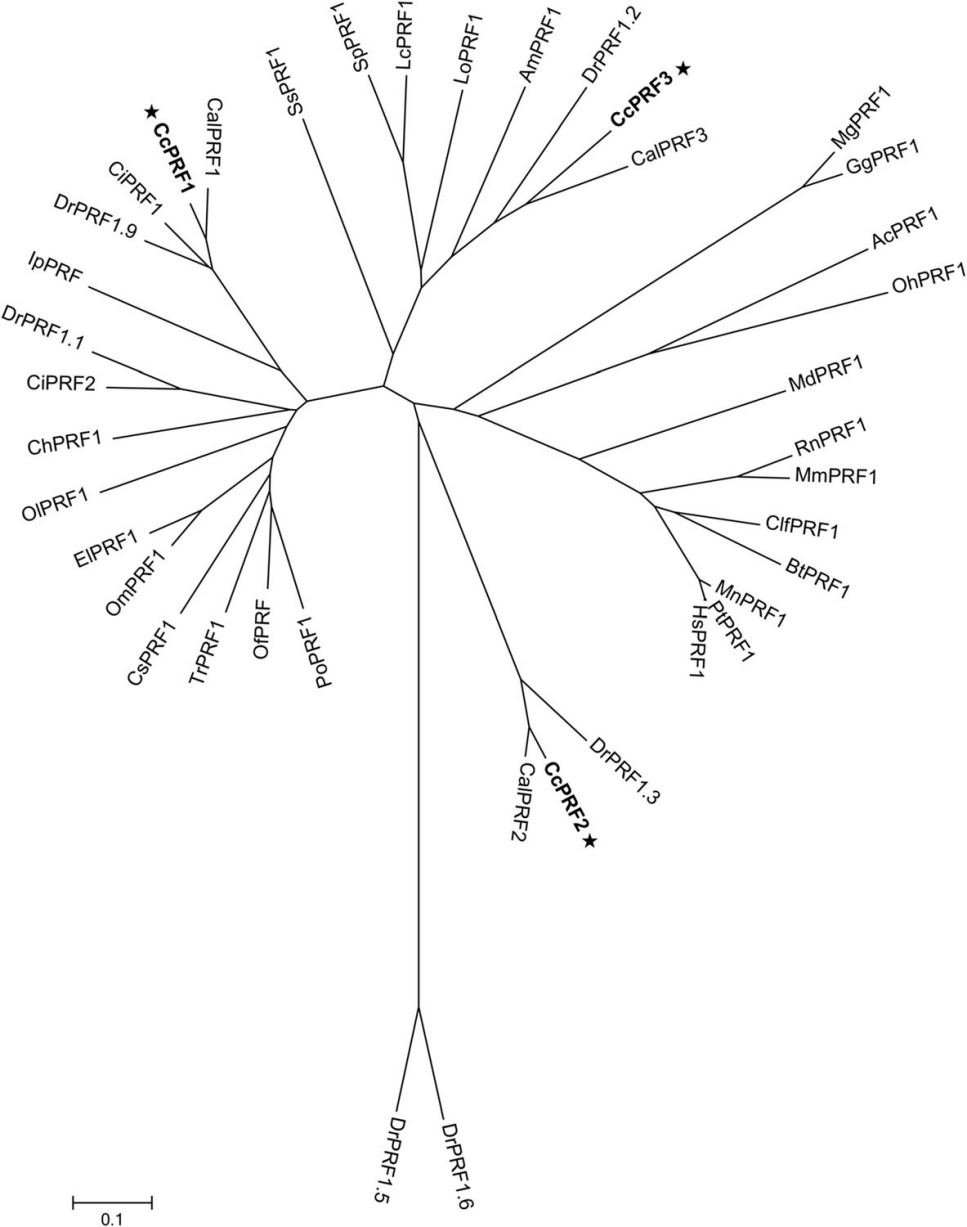
Phylogenetic analysis of perforin amino acid sequences. Phylogenetic tree is generated using the neighbor-joining (NJ) method in MEGA 6.0, and the GenBank accession numbers used are shown in Additional file 4: Table S4. Hs (*Homo sapiens*)**,** Pt (*Pan troglodytes*), Mn (*Macaca nemestrina*), Rn (*Rattus norvegicus*), Md (*Monodelphis domestica*), Mm (*Mus musculus*), Bt (*Bos Taurus*), Clf (*Canis lupus familiaris*), Gg (*Gallus gallus*), Mg (*Meleagris gallopavo*), Oh (*Ophiophagus hannah*), Ac (*Anolis carolinensis*), El (*Esox lucius*), Lo (*Lepisosteus oculatus*), Am (*Astyanax mexicanus*), Sp(*Stegastes partitus*), Ol (*Oryzias latipes*), Tr (*Takifugu rubripes*), Om (*Oncorhynchus mykiss*), Lc (*Larimichthys crocea*), Cs (*Cynoglossus semilaevis*), Cal (*Carassius auratus langsdorfii*), Ci (*Ctenopharyngodon idella*), Po (*Paralichthys olivaceus*), Of (*Oplegnathus fasciatus*), Dr. (*Danio rerio*), Ch (*Clupea harengus*), Ss (*Salmo salar*), Ip (*Ictalurus punctatus*)

Phylogenetic analysis of the MACPF family proteins (perforin, C6, C7, C8, C9) and the MACPF-domain containing protein (macrophage-expressed gene 1, Mpeg-1) showed that the three *Cc*PRFs and the perforins of other species were clustered in one branch, where fish perforins were separated from the cluster of other species, including the mammals, birds and amphibians (Additional file 9: Figure S1). While for common carp, the three *Cc*PRFs belonged to three different sub-branches, in which each *Cc*PRF was phylogenetically closest to the perforins of other cyprinid fish, such as the ginbuna crucian carp, zebrafish and grass carp (Fig. 3).

Homology analysis showed that *Cc*PRF1 shared the highest amino acid identities with crucian carp PRF1 (86.9%), grass carp PRF1 (83%) and zebrafish PRF1.9 (81%), *Cc*PRF2 with ginbuna crucian carp PRF2 (89.8%) and zebrafish PRF1.3 (76.6%), and *Cc*PRF3 with ginbuna crucian carp PRF3 (77.8%) and zebrafish PRF1.2 (71.0%). The amino acid identities of three *Cc*PRFs with the human, mouse, chicken and green anole perforin proteins ranged from 29.2 to 36.8% (Table 2). Additionally, as shown in Fig. 1e, each *Cc*PRF had less than amino acid 40.0% identities (37.3–39.5%) with the other two isoforms.

**Table 2:** Percent identity of perforin proteins between common carp and other species

Species	Name	*Cc*PRF1	*Cc*PRF2	*Cc*PRF3
Human	PRF1	36.1	36.7	36.2
House mouse	PRF1	34.7	36.3	36.6
Chicken	PRF1	28.9	31.7	30.8
Green anole	PRF1	33.5	33.2	32.1
Fugu rubripes	PRF1	49.3	36.6	38.2
Japanese flounder	PRF1	51.0	37.8	38.0
Atlantic salmon	PRF1	41.1	36.3	43.3
Zebrafish	PRF1.9	81.0	39.0	39.5
	PRF1.2	38.0	37.1	71.0
	PRF1.3	36.2	76.6	36.7
Ginbuna crucian carp	PRF1	86.9	36.2	37.6
	PRF2	36.0	89.8	36.5
	PRF3	37.5	37.6	77.8
Grass carp	PRF1	83.0	37.9	38.4
	PRF2	53.7	35.6	38.9

### Tissue distribution of three *Cc*PRFs in healthy common carps

**Fig. 4:**
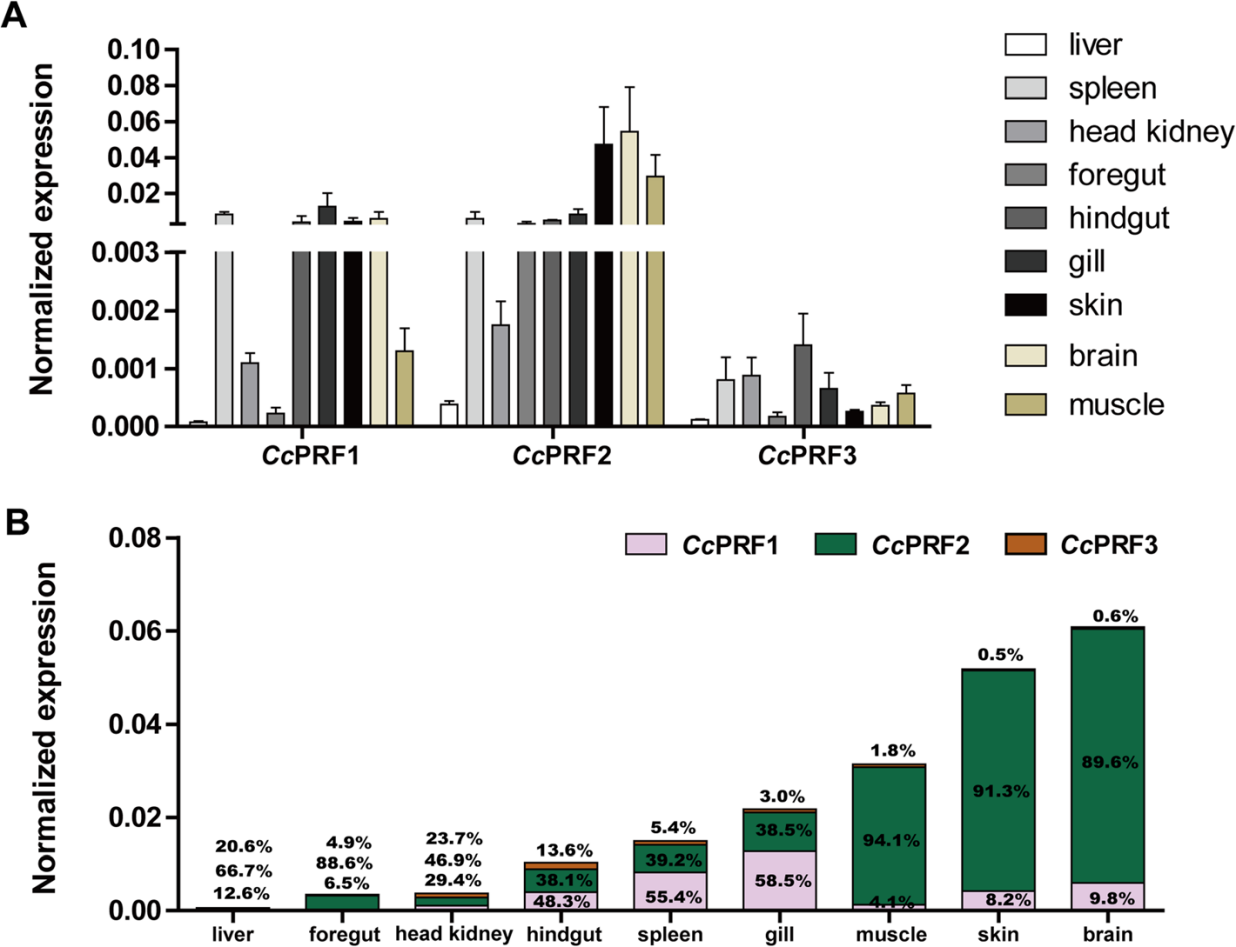
Constitutive tissue-specific expression of three *Cc*PRF genes in healthy common carps. **a** Relative expression of three *Cc*PRFs in the nine tissues (the liver, spleen, head kidney, foregut, hindgut, gill, skin, brain and muscle). **b** Cumulative expression and relative proportion (%) of three *Cc*PRFs in different tissues. In each tissue, the relative proportion of *Cc*PRF1 (bottom), *Cc*PRF2 (middle) and *Cc*PRF3 (top) were shown with percentage numbers. Relative gene expression level of each *Cc*PRF was detected using real-time PCR. Amplification of S11 in each tissue was performed as an internal control (*n* = 4)

Each *Cc*PRF was expressed in all the nine tested tissues, including the liver, spleen, head kidney, foregut, hindgut, gill, skin, brain and muscle, with different expression levels in various tissues (Fig. 4a). Moreover, in each tissue, the three *Cc*PRFs also showed differential expression profiles (Fig. 4b).

Specifically, the highest expression of *Cc*PRF1 mRNA was detected in the spleen, hindgut, gill, skin and brain, followed by the head kidney and muscle, and very low expression in the foregut and liver. *Cc*PRF2 was mainly distributed in the skin, brain and muscle, with a moderate level of expression observed in the spleen, foregut, hindgut and gill, while low gene expression in the head kidney and liver. For *Cc*PRF3, the mRNA expression was predominantly detected in the hindgut, spleen, head kidney, gill and muscle, followed by the brain and skin, and the lowest level in the foregut and liver.

Overall, in healthy carps, gene expression of *Cc*PRF2 predominated in most of the tested tissues, including the muscle (94.1%), skin (93.1%), brain (89.6%), foregut (88.6%), liver (66.7%), hindgut (48.3%) and head kidney (46.9%). In the spleen and gill, *Cc*PRF1 mRNA levels was the highest. In contrast, *Cc*PRF3 mRNA expression was obviously lower than *Cc*PRF1 and *Cc*PRF2, with the proportion of 23.7%, 13.6%, 5.4%, 4.9%, 3.0%, 1.8%, 0.5% and 0.6% in the head kidney, hindgut, spleen, foregut, gill, muscle, skin and brain, respectively. Moreover, in the nine tested tissues, the brain and skin showed the highest cumulative expression levels of the three *Cc*PRFs.

### Ontogeny of three *Cc*PRF genes in common carp larvae

**Fig. 5:**
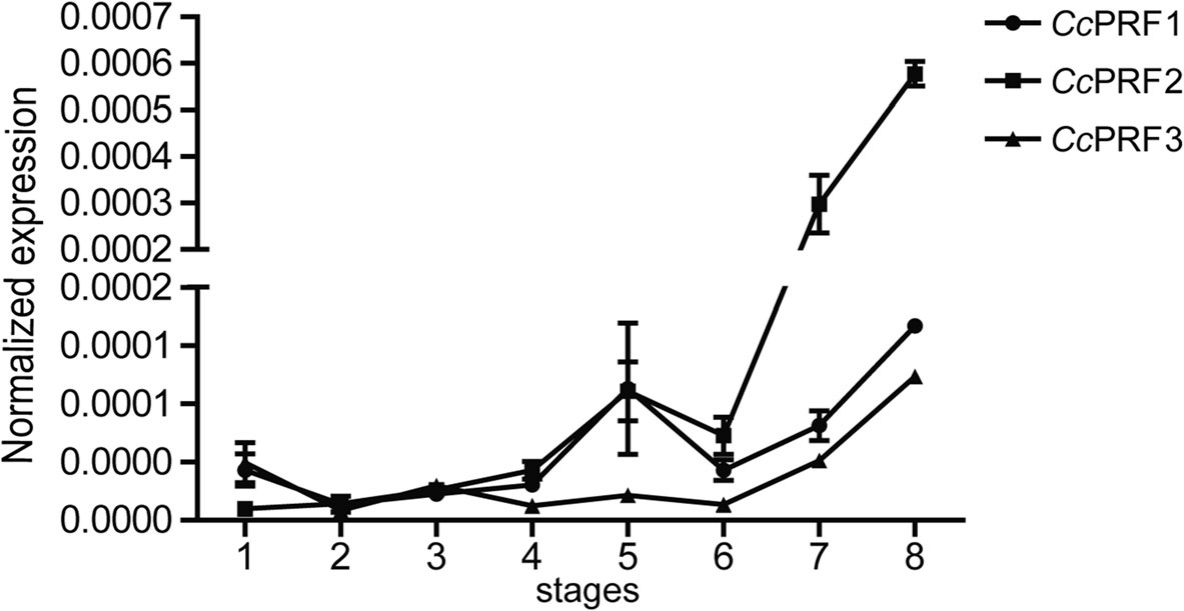
The mRNA expression patterns of three *Cc*PRF genes during larvae ontogeny. Each *Cc*PRF is shown with a distinct line graph. Abscissa values (1–8) are indicated as 8 developmental stages with (1) blastula stage; (2) optic primordium appearance; (3) 1 dph; (4) 2 dph; (5) 4 dph; (6) 8 dph; (7) 14 dph; (8) 22 dph. The relative expression of each *Cc*PRF is normalized to S11 gene and displayed as the means ± SD (*n* = 5)

As shown in Fig. 5, we analysed gene expression patterns of the three *Cc*PRFs from blastula stage to 22 dph. The results showed that *Cc*PRF1 had two expression peaks, with the first at 4 dph and the second at 22 dph. As for *Cc*PRF2, after fertilization, its gene expression was up-regulated at embryonic stage and continued to increase up to 22 dph. While for *Cc*PRF3, it displayed one expression peak at 22 dph. Obviously, the three *Cc*PRFs displayed differential expression patterns in larvae ontogeny, while with a common expression peak at 22 dph.

### Expression profiles of three *Cc*PRFs upon polyI:C and *A. hydrophila* in vivo

**Fig. 6:**
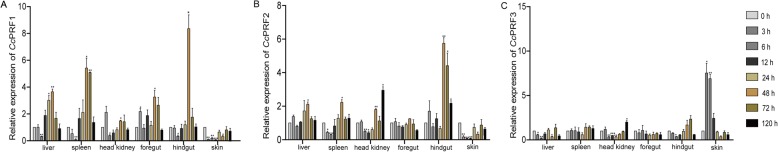
Gene expression of three *Cc*PRF genes after polyI:C stimulation in vivo. *Cc*PRF1, *Cc*PRF2 and *Cc*PRF3 are shown in **a**, **b** and **c**, respectively. The results are normalized to S11 gene. Data are presented as a fold change of the stimulated group to the un-stimulated group (denoted by 0 h) and displayed as the means ± SD (*n* = 3). *p < 0.05, **p < 0.01 and ***p < 0.001

After polyI:C stimulation, *Cc*PRF1 gene expression was detected highest at 2 dpi in the liver, spleen, head kidney, foregut and hindgut, with 3.7-fold, 5.4-fold, 1.5-fold, 3.2-fold and 8.3-fold higher expression than the control group, respectively (Fig. 6a, *p* < 0.05 or *p* < 0.01). *Cc*PRF2 mRNA level reached peak values at 2 dpi in the liver, spleen and hindgut, with 2.1-fold, 2.2-fold and 5.8-fold up-regulation, respectively, as compared with the control group (Fig. 6b, *p* < 0.05 or *p* < 0.01). In the head kidney, mRNA expression of *Cc*PRF2 began to increase at 2 dpi and reached a peak at 5 dpi (2.9-fold, *p* < 0.05). With regard to *Cc*PRF3, the mRNA expression of *Cc*PRF3 never increased significantly in the analysed tissues, with the exception of the skin (7.5-fold, *p* < 0.05) and head kidney (2.0-fold, *p* < 0.05) (Fig. 6c). Additionally, in the skin, *Cc*PRF1 and *Cc*PRF2 mRNA levels decreased significantly within 12hpi, and soon afterwards return to the basic expression level (Fig. 6a, b).

**Fig. 7:**
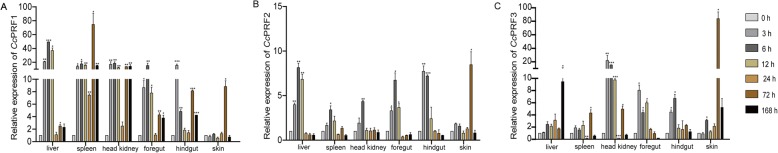
Gene expression of three *Cc*PRF genes after *A. hydrophila* stimulation in vivo. *Cc*PRF1, *Cc*PRF2 and *Cc*PRF3 are shown in **a**, **b** and **c**, respectively. The results are normalized to S11 gene. Data are presented as a fold change of the stimulated group to the un-stimulated group (denoted by 0 h) and displayed as the means ± SD (n = 3). *p < 0.05, **p < 0.01 and ***p < 0.001

After *A. hydrophila* stimulation, mRNA level of each *Cc*PRF was significantly up-regulated in all the analysed tissues, with large fold-change higher expression than that in the control (Fig. 7a, b, c, *p* < 0.05, *p* < 0.01 or *p* < 0.005). Moreover, similar to polyI:C-stimulated group, the three *Cc*PRFs showed different expression patterns in *A. hydrophila*-stimulated group*.*

### Expression profiles of *Cc*PRFs and CD8α in response to polyI:C and LPS in vitro

To further identify the immunologic function of perforin in common carp, we assayed gene expression of three *Cc*PRFs and CD8α upon polyI:C and LPS in PBLs and HKLs.

**Fig. 8:**
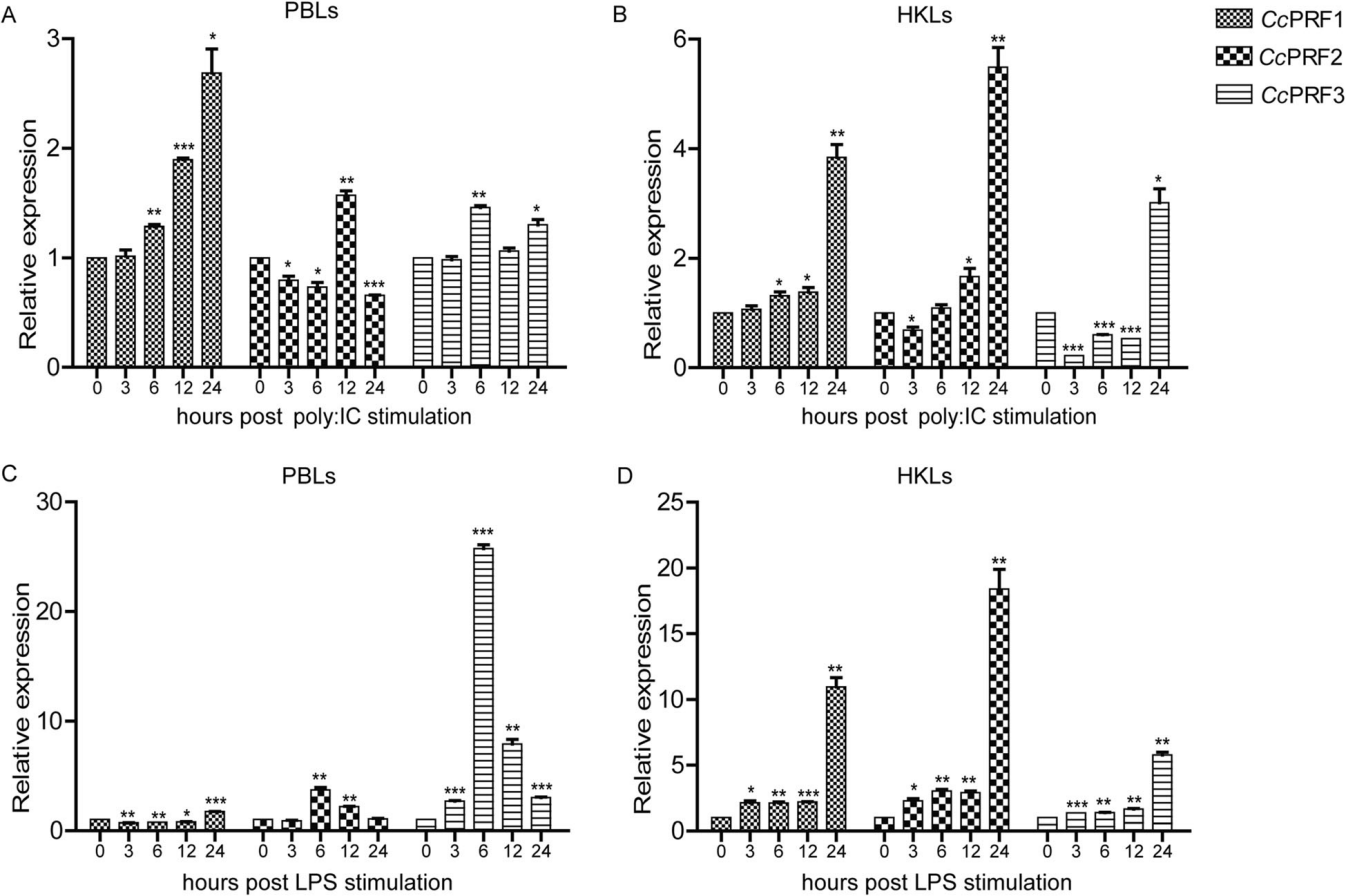
Gene expression of three *Cc*PRF genes after polyI:C and LPS stimulation in vitro. **a** Induced expression of the *Cc*PRFs by polyI:C in PBLs. **b** Induced expression of the *Cc*PRFs by polyI:C in HKLs. **c** Induced expression of the *Cc*PRFs by LPS in PBLs. **d** Induced expression of the *Cc*PRFs by LPS in HKLs. Three groups of PBLs or HKLs were treated with polyI:C or LPS independently. The results are normalized to S11 gene. Data are presented as a fold change of the stimulated group to the un-stimulated group (denoted by 0 h) and displayed as the means ± SD (n = 3). *p < 0.05, **p < 0.01 and ***p < 0.001

In PBLs, after polyI:C stimulation, mRNA expression of *Cc*PRF1, *Cc*PRF2 and *Cc*PRF3 reached peak values at 24 hpi, 12 hpi and 6 hpi, respectively, with 2.7-fold, 1.5-fold and 1.4-fold up-regulation, respectively, as compared with the control group (Fig. 8a, *p* < 0.05 or *p* < 0.01). In response to LPS, *Cc*PRF1 mRNA level reached the highest level at 24 hpi with 1.7-fold higher expression than the control group (Fig. 8c, *p* < 0.001). Gene expression of *Cc*PRF2 and *Cc*PRF3 was up-regulated to the highest level at 6 hpi, with a 3.7-fold and 25.2-fold higher expression than that of the control group, respectively (Fig. 8c, *p* < 0.01 or *p* < 0.005). In HKLs, gene expression of *Cc*PRF1, *Cc*PRF2 and *Cc*PRF3 was up-regulated by polyI:C and increased to peak values at 24 hpi, with 3.8-fold, 5.5-fold and 3.0-fold higher expression than the control (Fig. 8b, *p* < 0.01 or *p* < 0.005). Upon LPS stimulation, *Cc*PRF1, *Cc*PRF2 and *Cc*PRF3 mRNA expression reached the highest level at 24 hpi as well, with 11.0-fold, 18.4-fold and 5.8-fold higher expression than the control group (Fig. 8d, *p* < 0.005).

**Fig. 9:**
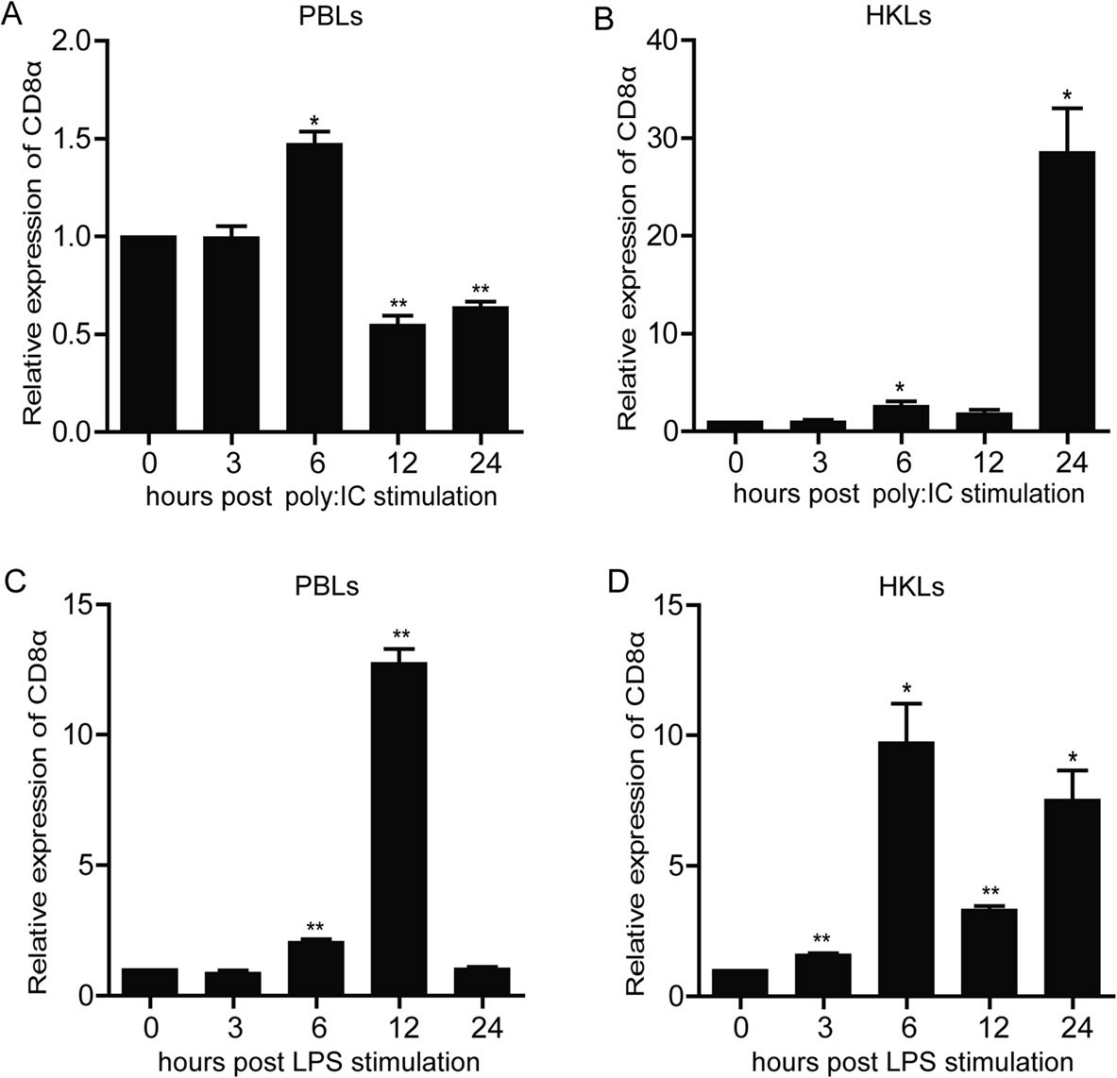
Gene expression of CD8α after polyI:C and LPS stimulation in vitro. **a** Induced expression of CD8α by polyI:C in PBLs. **b** Induced expression of CD8α by polyI:C in HKLs. **c** Induced expression of CD8α by LPS in PBLs. **d** Induced expression of CD8α by LPS in HKLs. Three groups of PBLs were treated with polyI:C or LPS independently. The results are normalized to S11 gene. Data are presented as a fold change of the stimulated group to the un-stimulated group (denoted by 0 h) and displayed as the means ± SD (n = 3). *p < 0.05, **p < 0.01 and ***p < 0.001

As for CD8α, in PBLs, polyI:C induced the gene expression of CD8α, with 1.5-fold (6 hpi) higher expression than the control group (Fig. 9a, *p* < 0.05). In response to LPS, CD8α mRNA expression was increased significantly, with 12.8-fold (12 hpi) higher expression than the control group (Fig. 9c, *p* < 0.01). While in HKLs, upon polyI:C challenge, the gene expression of CD8α reached the highest level at 24 hpi, in which time point the expression level was 28.6-fold higher than the control (Fig. 9b, *p* < 0.05). In response to LPS, CD8α mRNA levels reached the highest at 6 hpi, with 9.7-fold higher expression than the control (Fig. 9d, *p* < 0.05).

## Discussion

In the present study, we cloned and confirmed three perforin isoform genes from common carp, named *Cc*PRF1, *Cc*PRF2 and *Cc*PRF3. The three *Cc*PRFs were quite different with each other, with nucleic acid identities of ORF ranged from 47.0 to 47.9% and amino acid identities from 37.3 to 39.5% (Fig. 1e). Nevertheless, in the phylogenetic tree of perforin, C6, C7, C8, C9 and Mpeg-1, the same evolutionary branch of *Cc*PRFs with other species’ perforins strongly implied that the three *Cc*PRFs all belong to the perforin superfamily (Additional file 9: Figure S1). The three *Cc*PRF proteins were all comprised of a signal peptide, a middle MACPF domain and a C-terminal CalB domain, exhibiting the same domain architecture as mammalian orthologs [34, 35] although the three *Cc*PRFs only shared 34.7–36.7% amino acid identities with human and mouse perforins (Table 2). Thus, the consistency of *Cc*PRF protein domains with the typical structure of perforin protein implied that *Cc*PRFs might play the similar role as do the other species’ perforins [36].

The intron-exon arrangement of perforin gene in humans, mice and rats was conserved, in which they were all comprised of three exons and two introns, and the sizes of the exons located in their ORFs were 536/539 and 1129 bp (Fig. 2). However, these characteristics were quite different from that of the three *Cc*PRF genes and other fish perforins, which was in line with the previous suggestion of different genomic structures of the perforin between teleosts and mammals [16]. On the other hand, for *Cc*PRF1, its intron-exon arrangement was similar to that of zebrafish PRF1.9 and grass carp, channel catfish and Japanese flounder PRF1, particularly showing more similarities with grass carp, channel catfish and zebrafish, with the same exon sizes (530, 139, 218, 880), than Japanese flounder. *Cc*PRF2 genomic DNA comprised five exons and four introns, and the sizes of the exons located in its ORF were 503, 271, 599 and 304. Similarly, for zebrafish PRF1.3 gene, the size of the first exon located in its ORF was 503 bp as well. And for *Cc*PRF3, the size of the last exon located in ORF was 1207 bp, which was the same as that of zebrafiah PRF1.2 gene. Notably, all these similarities were in line with the phylogenetic relationship of perforin in fish species (Fig. 3). Moreover, although intron/exon arrangement of zebrafish PRF1.5 and PRF1.6 was largely different form that of the three *Cc*PRFs, the exons of these two zebrafish perforins had highly similarities with each other, which was in accordance with their very close evolutionary relationship. Thus, taken together all these results implying that intron-exon organization of perforin gene was in accordance with the evolutionary relationship and species evolution.

Similar to the previous report that under basal conditions 6 zebrafish perforin genes were all expressed in the liver, spleen, kidney, intestine, gill and muscle [22]. Each *Cc*PRF gene was expressed in the nine analysed tissues (the liver, spleen, head kidney, foregut, hindgut, gill, skin, brain and muscle) (Fig. 4). However, it was reported that perforin gene was not able to be detected in the liver, stomach, brain, muscle, fin and eye of Japanese flounder [15, 16] and the liver of trout [18]. Although the tissue distribution pattern of perforin was inconsistent in fish species, its gene expression was always detected in the pivotal immune-related tissues (the head kidney, spleen, intestine, gill and skin), at least implying the potential role of fish perforin in the immune system.

Previously, it was shown that the innate immune system is a fundamental defense weapon of fish during early stages of development [37–39]. In the common carp, it was reported that innate immune factors such as C3 and α2-macroglobulin were present before hatching and these factors were involved in the immune defense response during ontogeny of carp [38, 40]. Additionally, in the zebrafish, it was demonstrated that immune system of the larvae was completely mature between 4 and 6 weeks post fertilization [41]. In accordance with this time period, during the larvae ontogeny of zebrafish, expression peaks were observed around the fourth week of development (23 dpf and 29 dpf) in several perforin genes [22]. Similarly, in the early development stages of common carp larvae, in spite of differential expression patterns for the three *Cc*PRFs, they showed a common expression peak at 22 dph (Fig. 5). Moreover, in the larval development of Japanese flounder, perforin gene was significantly expressed during nephron formation [15]. Thus, all the results indicated that together with other immune-related factors, fish perforin may play a crucial role in the larval immune system.

PolyI:C is a mimetic of virus-derived double-stranded RNA, and is usually designed to mimic viral infections [42, 43]. In mammals, polyI:C or virus was able to induce the expression of perforin in cytotoxic cells. In the mouse hepatic and splenic NK cells [44, 45] and human NK cells isolated from peripheral blood [46], stimulation with polyI:C increased gene expression or protein secretion of the perforin. Using perforin-deficient mice, it was demonstrated that CD8^+^ T cell-mediated protection against viral infection, such as Ebola virus, West Nile virus and influenza virus, was dependent on perforin [47–49]. In addition, in fish, some studies have reported gene expression of perforin in response to viral stimulation. In the kidney of rock bream injected with rock bream iridovirus (RBIV) or megalocytivirus, significant up-regulation of perforin gene expression was observed at 4 or 8 dpi [20, 21]. In the rainbow trout RTS11 cell line, infection with viral hemorrhagic septicaemia virus (VHSV) up-regulated perforin gene expression [50]. In the zebrafish kidney, in vivo stimulation with spring viremia of carp virus (SVCV) increased mRNA levels of multiple perforins including PRF1.1, 1.3, 1.5, 1.6 and 1.9 at 6 hpi, and polyI:C induced PRF1.6 expression at 3 hpi [22]. Similarly, in common carp, mRNA levels of the three *Cc*PRFs were significantly up-regulated in response to polyI:C in vivo and in vitro (Figs. 6 and 8a, b). However, in contrast to SVCV, polyI:C was not able to increase mRNA levels of zebrafish perforins within 24 hpi, with the exception of PRF1.6, in the kidney [22], which was consistent with the in vivo results in common carp. Additionally, rock bream perforin gene was not induced by virus shortly post infection, until a few days later [20, 21]. Moreover, unlike trout RTS11, in the trout HKLs, no significant up-regulation of perforin expression was observed after incubation for 24 h with VHSV [50]. These data suggested that perforin expression was induced by viral stimulation possibly in a time- and cell population- dependent manner, which may partly explain why in common carp the induction of perforin by polyI:C had the relative tissue-specificity. Nevertheless, in PBLs and HKLs, three *Cc*PRF mRNAs were all up-regulated by polyI:C (Fig. 8a, b). Collectively, all these data implied that perforin might play significant roles in the protective host immune response of common carp to virus.

To date only few studies investigated the modulation of perforin gene expression in response to bacterial pathogens, especially the extracellular bacteria. In human NK cells, extracellular mycobacteria induced mRNA and protein production of the perforin, which eliminated the bacteria by damaging cell wall structures [3]. In the fish rohu, stimulation with Gram-negative bacterium *A. hydrophila* significantly up-regulated expression of perforin gene, particularly in the spleen and gill tissues [51]. Additionally, in the sponge, a cDNA clone resembling human perforin displayed strong antibacterial activaty [52]. Here, in common carp, after stimulation of *A. hydrophila*, mRNA levels of the three *Cc*PRFs was remarkably enhanced in a variety of immune-related tissues (Fig. 7). Meanwhile, in vitro, stimulation with LPS also increased gene expression of the carp perforins (Fig. 8c, d). Similarly, in the zebrafish adult kidney, significant up-expression of PRF1.1, 1.2, 1.6 and 1.9 was observed over the time course post injection with LPS [22]. LPS is a major component shared in the outer membrane of Gram-negative bacteria, and previous reports showed that LPS was the main virulence factor responsible for the dominant response to Gram-negative bacteria infection [53, 54]. So, based on these data indicating possible relevance of LPS in *A. hydrophila*-induced up-regulation of *Cc*PRFs. Taken together, all these results suggested potential involvement of perforin in the immune defense of common carp against bacteria.

In mammals, polyI:C or virus was capable of augmenting antigen-specific responses in CD8^+^ T cells, with inducing perforin expression [55–58]. Moreover, in fish species, a recruitment of CD8α^+^ cells was found in the liver of rainbow trout in response to VHSH, meantime with an up-regulation of CD8α and perforin genes observed [59]. Similarly, in rock bream, upon RBIV infection, CD8α gene expression was increased, accompanied by the induction of perforin at the same time point [21]. As for LPS, via an indirect pathway involving antigen presenting cells (APCs), it is able to promote proliferation and activation of murine CD8^+^ T cells [60, 61]. Additionally, in humans, LPS could directly activate naive CD8^+^ T cells via TLR4 on their cell membranes, and afterwards these activated cells secreted perforin in response to LPS [2]. In line with these findings, in common carp, gene expression of CD8α (a main marker of teleost CTLs [62]) was increased by polyI:C and LPS in vitro, coincident with the induced expression pattern of perforin. So, based on all these observations, it was inferred that, in common carp, polyI:C and LPS may induce immune activation of CD8^+^ T cells, after which this type of cells may, at least in part, contribute to the production of perforin in response to immune stimulation.

Besides, it was worth noting that tissue distribution pattern was different for each *Cc*PRF isoform, where *Cc*PRF2 showed broad expression across various tissues, and the other two were more tissue-specific. Additionally, differential expression dynamics were also detected during the larvae ontogeny of common carp (Fig. 5) and in response to immune stimulation (Figs. 6 and 7). As previously reported, the similar phenomenon has been observed in other fish species. In the zebrafish, 6 identified perforin isoforms had diverse constitutive expression patterns in healthy organisms and in larvae ontogeny. And as mentioned above, in the zebrafish kidney, 6 perforins displayed differential response dynamics upon LPS, polyI:C and SVCV [22]. Moreover, in crucian carp CTLs from allo-sensitized fish kidneys, PRF1 mRNA was increased, while PRF2 and PRF3 not changed significantly [19]. These observations raise an interesting question of why fish possess multiple perforin isoforms with different expression dynamics. One explanation is that fish perforins have broader cell distribution and roles or more complex regulatory patterns than in mammals. For instance, perforin is essentially restricted to NK cells and CTLs in mammals, while in fish perforin may be present in more types of immune cells with each isoform being restricted to a particular type. Alternatively, all perforin isoforms may be present in the same cell type, with each responding to distinct signals. Actually, in one previous study, it has been found that multiple isoforms of fish perforins were present at distant loci [63], implying that these perforin genes were likely to be controlled by different regulatory elements. Thus, these facts implied possible different cell locations or functional differences for multiple fish perforin genes, which probably evolved as a strategy to expand immune recognition capabilities in fish and compensate for their limitations of adaptive immunity [64].

## Conclusions

In the present study, three perforin isoform genes, named *Cc*PRF1, *Cc*PRF2 and *Cc*PRF3, were identified and investigated for the first time in common carp. Evolution analysis shows that *Cc*PRF genes have closer evolutionary relationship with other fish perforins than those of mammals, in regard to both amino acid identities and genomic organization. The constitutive expressions of three *Cc*PRFs in early life stages of development imply possible relevance of perforin to the immune system of common carp larvae. Moreover, the significant up-regulation of multiple *Cc*PRF genes indicate that perforin might play significant roles in the immune defense of common carp against viral and bacterial pathogens. Meanwhile, the differential expression dynamics were observed across three *Cc*PRF genes, implying possible different cellular locations or functional differences for various perforin isoforms. These observations could provide some clues for preventing carp infection by pathogenic microorganisms present in the aquatic environment.

## Additional files

Additional file 1: **Table S1.** Primers used for cDNA cloning. (DOCX 15 kb)

Additional file 2: **Table S2.** Primers used for genomic DNA cloning. (DOCX 14 kb)

Additional file 3: **Table S3.** Primers used for Real-time PCR. (DOCX 14 kb)

Additional file 4: **Table S4.** GenBank accession numbers for perforin proteins. (DOCX 17 kb)

Additional file 5: **Table S5.** GenBank accession numbers of Mpeg-1, C6, C7, C8 and C9 proteins. (DOCX 15 kb)

Additional file 6: Intron and exon sequences of *Cc*PRF1. (DOCX 13 kb)

Additional file 7: Intron and exon sequences of *Cc*PRF2. (DOCX 14 kb)

Additional file 8: Intron and exon sequences of *Cc*PRF3. (DOCX 13 kb)

Additional file 9: **Figure S1.** Phylogenetic analysis of perforin, Mpeg-1, C6, C7, C8 and C9 polypeptides. This tree is generated using the neighbor-joining (NJ) method in MEGA 6.0. GenBank accession numbers used are shown in Additional file 4: Tables S4 and Additional file 5: Table S5, and the species abbreviations in Fig. 3. (TIF 29791 kb)

Additional file 10: ARRIVE (Animal Research: Reporting of in Vivo Experiments) checklist.

## Abbreviations

*A. hydrophila*: *Aeromonas hydrophila*
ANOVA: analysis of variance
APCs: antigen presenting cells
CalB: calcium-binding
CTLs: cytotoxic T lymphocytes
dpf: days post fertilization
dph: days post hatching
EGF: epidermal growth factor
FCS: fetal calf serum
Gzm: granzyme
HKLs: head kidney leukocytes
hpi: hours post injection
JTT: Jones-Taylor-Thornton
LPS: lipopolysaccharides
MACPF: membrane-attack-complex/perforin
Mpeg-1: macrophage-expressed gene 1
NK: natural killer
ORF: open reading frame
PBLs: peripheral blood leukocytes
PBS: phosphate-buffered saline
PolyI:C: polyinosinic-polycytidylic acid
RACE: rapid amplification of the cDNA ends
RBIV: rock bream iridovirus
SMART: simple modular architecture research tool;
SVCV: spring viremia of carp virus
UTR: untranslated region
VHSV: viral hemorrhagic septicaemia virus
