# Supplementary material for: Molecular characterization of three novel perforins in common carp (Cyprinus carpio L.) and their expression patterns during larvae ontogeny and in response to immune challenges
Source: BMC Vet Res. 2018 Oct 3;14:299. doi: 10.1186/s12917-018-1613-y (PMC6169072; doi:10.1186/s12917-018-1613-y)
Supplement: Supplementary file 1 — Table S1. Primers used for cDNA cloning. (DOCX 15 kb) [file 12917_2018_1613_MOESM1_ESM.docx]

**Additional file 1: Table S1 Primers used for cDNA cloning.**

| Name | Sequence (5’-3’) | Application | Annealing temperature (°C) |
| --- | --- | --- | --- |
| PRF1-F | TTACGCCGAGCCATTCGTGACT | cDNA amplification | 60 |
| PRF1-R | GTCATCGTCCCACTTGTTGTC |  | 60 |
| PRF1-GSP-5inner | TGTTATGACAGGTGCACACACAG | Nested 5'-RACE PCR | 59 |
| PRF1-GSP-5outer | TTTCTCAGGACCTTGACATAGCC | First round 5'-RACE | 59 |
| PRF1 -GSP-3innerr | GGACAACAAGTGGGACGATGAC | Nested 3'-RACE PCR | 58 |
| PRF1-GSP-3outer | ACTCACCAACCTGGAACTGGAAT | First round 3'-RACE | 58 |
| PRF2-F | GACACACTTCTTGCGGAGAGTCGA | cDNA amplification | 60 |
| PRF2-R | ACCATCCGTTTTAGAGAAATAGTC |  | 60 |
| PRF2-GSP-5inner | GTCTTCACAGCAGTGGTTGAATGC | Nested 5'-RACE PCR | 61 |
| PRF2-GSP-5outer | GACAGACCCTTCATGGAAACCTGG | First round 5'-RACE | 61 |
| PRF2-GSP-3innerr | CTGTGTTGAGAGGTGCAGGATTAT | Nested 3'-RACE PCR | 59 |
| PRF2-GSP-3outer | CAAGTAATCCAGGAGTGGCCACAT | First round 3'-RACE | 59 |
| PRF3-F | AGCTACCAGCAGCAGTTGTAGACT | cDNA amplification | 60 |
| PRF3-R | TCTTGAGCTGAAAATATCAGCCAG |  | 60 |
| PRF3-GSP-5inner | GACTGGAGACTTTCATTGAACAC | Nested 5'-RACE PCR | 58 |
| PRF3-GSP-5outer | CCTACTTTCCAACCATCTGGGAGT | First round 5'-RACE | 58 |
| PRF3-GSP-3innerr | AAACCTTCTCCAATGGCTGCCCAT | Nested 3'-RACE PCR | 59 |
| PRF3-GSP-3outer | AGTCTGCAGGGCCCACAGTGTAAT | First round 3'-RACE | 59 |
