# Supplementary material for: Molecular characterization of three novel perforins in common carp (Cyprinus carpio L.) and their expression patterns during larvae ontogeny and in response to immune challenges
Source: BMC Vet Res. 2018 Oct 3;14:299. doi: 10.1186/s12917-018-1613-y (PMC6169072; doi:10.1186/s12917-018-1613-y)
Supplement: Supplementary file 2 — Table S2. Primers used for genomic DNA cloning. (DOCX 14 kb) [file 12917_2018_1613_MOESM2_ESM.docx]

**Additional file 2: Table S2 Primers used for genomic DNA cloning.**

| Name | Sequence(5’-3’) | Annealing temperature (°C) |
| --- | --- | --- |
| PRF1-gF1 | GAAATCAGTCTGTGTTGGGCGAT | 59 |
| PRF1-gR1 | AGCCAACATCACCGACATCTTAC | 59 |
| PRF1-gF2 | GTAAGATGTCGGTGATGTTGGCT | 58 |
| PRF1-gR2 | TGACGGTATGCCGTGTCTATGCT | 58 |
| PRF1-gF3 | AGCATAGACACGGCATACCGTCA | 60 |
| PRF1-gR3 | GTGCTTTGATGATAAGCTTTCACA | 60 |
| PRF2-gF1 | AAGACTGTTAAGAAACGTCTCAA | 57 |
| PRF2-gR2 | AGCTGGCCGATTCGTAAATCTTG | 57 |
| PRF2-gF2 | CAAGATTTACGAATCGGCCAGCT | 59 |
| PRF2-gR2 | ACTCTCCGCAAGAAGTGTGTCCC | 59 |
| PRF2-gF3 | GGGACACACTTCTTGCGGAGAGT | 60 |
| PRF2-gR3 | GTCAATGTGGCCACTCCTGGATT | 60 |
| PRF2-gF4 | AATCCAGGAGTGGCCACATTGAC | 59 |
| PRF2-gR4 | TCTCAATATGGTGGTTATAGTATAC | 59 |
| PRF3-gF1 | TCATATGAAATGAGAAGTTAGAGAG | 60 |
| PRF3-gR2 | CGTAATTTACAAGTGCCATTTCCT | 60 |
| PRF3-gF2 | AGGAAATGGCACTTGTAAATTACG | 57 |
| PRF3-gR2 | TCTGTGAACCAGAAGCTTCCACAT | 57 |
| PRF3-gF3 | ATGTGGAAGCTTCTGGTTCACAGA | 58 |
| PRF3-gR3 | TATTAATGCAAGAACGCAGAAAGG | 58 |
