# Supplementary material for: Molecular characterization of three novel perforins in common carp (Cyprinus carpio L.) and their expression patterns during larvae ontogeny and in response to immune challenges
Source: BMC Vet Res. 2018 Oct 3;14:299. doi: 10.1186/s12917-018-1613-y (PMC6169072; doi:10.1186/s12917-018-1613-y)
Supplement: Supplementary file 3 — Table S3. Primers used for Real-time PCR. (DOCX 14 kb) [file 12917_2018_1613_MOESM3_ESM.docx]

**Additional file 3: Table S3 Primers used for Real-time PCR.**

| Name | Sequence (5’-3’) | [Annealing](javascript:void(0);) [temperature](javascript:void(0);) (°C) |
| --- | --- | --- |
| PRF1-F | GTGCTTTCAATCAGTGGCTGTC | 58 |
| PRF1-R | AGTATGTAGTCACGAATGGCTCG | 58 |
| PRF2-F | TGACTGTGTTGAGAGGTGCAGGA | 58 |
| PRF2-R | GTCTCATATGCACGGCTTCCGTAA | 58 |
| PRF3-F | CACTCCCAGATGGTTGGAAAGTAG | 58 |
| PRF3-R | TGCACCCTGCTTCATGTTTGGTG | 58 |
| CD8α-F | AAGGTTAGTTTGGCCATTCAGTC | 58 |
| CD8α-R | CAGTCAGCTCTCCAAAGATGAG | 58 |
| S11-F | ACGAGAGGGCTTATCAGAAGCAGC | 58 |
| S11-R | AGCCCAAGCCAACGTTTCGGTGAT | 58 |
