# Supplementary material for: Molecular characterization of three novel perforins in common carp (Cyprinus carpio L.) and their expression patterns during larvae ontogeny and in response to immune challenges
Source: BMC Vet Res. 2018 Oct 3;14:299. doi: 10.1186/s12917-018-1613-y (PMC6169072; doi:10.1186/s12917-018-1613-y)
Supplement: Supplementary file 4 — Table S4. GenBank accession numbers for perforin proteins. (DOCX 17 kb) [file 12917_2018_1613_MOESM4_ESM.docx]

**Additional file 4: Table S4 GenBank accession numbers for perforin** **proteins.**

| Species | Osmamthus fragrans and numbers | Protein length (aa) | GenBank accession no. |
| --- | --- | --- | --- |
| Human | *Homo sapiens* PRF1 | 555 | NP_005032 |
| Chimpanzee | *Pan troglodytes* PRF1 | 569 | XP_016773989 |
| Pig-tailed macaque | *Macaca nemestrina* PRF1 | 555 | NP_001292853 |
| Norway rat | *Rattus norvegicus* PRF1 | 554 | NP_059026 |
| Gray short-tailed opossum | *Monodelphis domestica* PRF1 | 559 | XP_001375097 |
| House mouse | *Mus musculus* PRF1 | 554 | [NP_035203](https://www.ncbi.nlm.nih.gov/protein/225735600) |
| Cattle | *Bos taurus* PRF1 | 554 | NP_001137207 |
| Dog | *Canis lupus familiaris* PRF1 | 555 | NP_001184111 |
| Chicken | *Gallus gallus* PRF1 | 644 | AGL75461 |
| Turkey | *Meleagris gallopavo* PRF1 | 641 | XP_010718967 |
| King cobra | *Ophiophagus hannah* PRF1 | 561 | ETE58488 |
| Green anole | *Anolis carolinensis* PRF1 | 585 | XP_008113574 |
| Northern pike | *Esox lucius* PRF1 | 589 | XP_010872443 |
| Spotted gar | *Lepisosteus oculatus* PRF1 | 577 | XP_006641735 |
| Mexican tetra | *Astyanax mexicanus* PRF1 | 575 | XP_007254774 |
| Bicolor damselfish | *Stegastes partitus* PRF1 | 570 | XP_008300135 |
| Japanese medaka | *Oryzias latipes* PRF1 | 597 | XP_011476115 |
| Fugu rubripes | *Takifugu rubripes* PRF1 | 597 | XP_003964971 |
| Rainbow trout | *Oncorhynchus mykiss* PRF1 | 589 | CAL29414 |
| Large yellow crocea | *Larimichthys crocea* PRF1 | 569 | KKF24071 |
| Tongue sole | *Cynoglossus semilaevis* PRF1 | 575 | XP_008327592 |
| Ginbuna crucian carp | *Carassius auratus langsdorfii* PRF1 | 587 | BAJ07835 |
|  | *Carassius auratus langsdorfii* PRF2 | 558 | BAJ07836 |
|  | *Carassius auratus langsdorfii* PRF3 | 568 | BAJ07837 |
| Grass carp | *Ctenopharyngodon idella* PRF1 | 588 | ABU96277 |
|  | *Ctenopharyngodon idella* PRF2 | 579 | ABU96278 |
| Japanese flounder | *Paralichthys olivaceus* PRF1 | 587 | BAC76420 |
| Barred knifejaw | *Oplegnathus fasciatus* perforin | 587 | AHH30804 |
| Zebrafish | *Danio rerio* PRF1.9 | 588 | ALA27409 |
|  | *Danio rerio* PRF1.1 | 580 | ALA27408 |
|  | *Danio rerio* PRF1.5 | 517 | ALA27407 |
|  | *Danio rerio* PRF1.6 | 516 | ALA27406 |
|  | *Danio rerio* PRF1.2 | 574 | ALA27405 |
|  | *Danio rerio* PRF1.3 | 555 | ALA27404 |
| Atlantic herring | *Clupea harengus* PRF1 | 582 | XP_012680058 |
| Atlantic salmon | *Salmo salar* PRF1 | 597 | ACI33854 |
| Channel catfish | *Ictalurus punctatus* perforin | 588 | AMM02738 |
