# Supplementary material for: Molecular characterization of three novel perforins in common carp (Cyprinus carpio L.) and their expression patterns during larvae ontogeny and in response to immune challenges
Source: BMC Vet Res. 2018 Oct 3;14:299. doi: 10.1186/s12917-018-1613-y (PMC6169072; doi:10.1186/s12917-018-1613-y)
Supplement: Supplementary file 5 — Table S5. GenBank accession numbers of Mpeg-1, C6, C7, C8 and C9 proteins. (DOCX 15 kb) [file 12917_2018_1613_MOESM5_ESM.docx]

**Additional file 5: Table S5 GenBank accession numbers of Mpeg-1, C6, C7, C8a, C8b and C9 proteins.**

| Species | Mpeg-1 | C6 | C7 | C8a | C8b | C9 |
| --- | --- | --- | --- | --- | --- | --- |
| Human | NP_001034485 | NP_000056 | NP_000578 | NP_000553 | NP_000057 | AAH20721 |
| Chimpanzee | NP_001009015 | NP_001009015 | JAA18409 | XP_016804717 | XP_009457007 | XP_009447516 |
| Norway rat | NP_001292389 | NP_788263 | XP_008759053 | NP_001100140 | NP_001178688 | NP_476487 |
| House mouse | NP_034951 | NP_057913 | NP_001230766 | NP_001277574 | NP_001303600 | NP_038513 |
| Cattle | AAI05416 | NP_001039444 | NP_001039431 | NP_001039750 | NP_001039606 | NP_001030441 |
| Chicken | XP_003641422 | NP_001138321 | NP_001305331 | XP_015146668 | NP_001308472 |  |
| Northern pike | XP_010875395 | XP_019909019 | XP_010890748 | XP_010885181 | XP_010885201 | XP_010901387 |
| Japanese medaka |  | XP_011480403 | XP_004072355 XP_011477267 | XP_004079021 | XP_004072355 | XP_004074560 |
| Fugu rubripes | XP_003974834  XP_011613879 | XP_011602776 | XP_003965188 | XP_003975886 | XP_003975877 | AAC60288 |
| Rainbow trout |  | NP_001118093 | NP_001117879  NP_001118090 | NP_001118096 | NP_001118079 | NP_001117898 |
| Large yellow crocea | XP_019115474 | KKF28531 | AJA33609 | XP_010746370 | XP_010746369 | KKF13134 |
| Grass carp |  | AFN26001 | AFN20333 |  |  | ABN49522 |
| Zebrafish | AAH55644 | NP_956932 | XP_005161294 | NP_001003496 | NP_001243652 | NP_001314855 |
| Atlantic salmon | XP_014027977 | NP_001167046 | NP_001133245 | XP_013995979 | XP_014047216 | XP_013994122 |
| Channel catfish |  | XP_017348510  XP_017348511 | XP_017348512 | XP_017328550 | XP_017328556 | XP_017315885 |
| Common carp |  |  |  | XP_018927492 | XP_018927491 | XP_018951266 |
