# Supplementary material for: Molecular characterization of three novel perforins in common carp (Cyprinus carpio L.) and their expression patterns during larvae ontogeny and in response to immune challenges
Source: BMC Vet Res. 2018 Oct 3;14:299. doi: 10.1186/s12917-018-1613-y (PMC6169072; doi:10.1186/s12917-018-1613-y)
Supplement: Supplementary file 6 — Intron and exon sequences of CcPRF1. (DOCX 13 kb) [file 12917_2018_1613_MOESM6_ESM.docx]

Exon 1

gaaatcagtctgtgttgggcgatcttagattacaattaaaaagacctcgcag

Exon 2

aactcgcaatgattcctctagttatcctgtggtctgggcttcttctgtaccttcctcctccaaccagcccggcatgctttctgggtaaagaagcagagtgtcgagatgcaaattttgtgccaggatctgacttggctggagagggtttcgacataactaagatgcaacgcctgggcacttttgtcatcgacatgagtaagtgggagctcaagaataacacatgtaacctgtgcaaaaacccattcatgcagaacaaaaagcagaagctcccggtgtctgtgctttactggagggccacccaaaaatgcaaaaggtcattatccagctccatttatgagtccagcgagtccctggtcagttccagcacctcctccgtagagaacaactggaaagtcggcctgggaataggcaacatggtaagtaagatgtcggtgatgttggctggaacgaactccagactcgctgagtattcaatggcaaagaccaaaaaggacaagttcagcttcatcaagcagtctacatcctgcggatactatgg

Exon 3

atacagaattagcagccgctctcctcttcatcacgagctaagttatgaattaaccacgcttccagaaacatatgacaaccaaacaaaagaaagctacttgtcacttattgaaaagtttggcacacattacactgtccat

Exon 4

gtgaaactgggtggaacagtgcaatctgtgaccagtgtcaaagaatgcatggctactctacaggacctcagtacggatgaggtcaaaacatgtctggacgtcgaggcatctgcaagtgtaatggggaggatcagcatagacacggcataccgtcactgtaaagagagcaaagacaaaaaactgagcaagcacagtttttctaacagttttactgacag

Exon 5

gttaactgaaatcacgggtggccatgctcaagacacagcgctcctcttctcagccagtaatgaccctggtgctttcaatcagtggctgtccacagtgccagaaaaaccagaaattatttccttttcactgaagcccctccatatgttgctgccagtcaaggacccaaaatatgagcagttacgccgagccattcgtgactacatactgcagagggccctttggaagaaatgctctacaccttgcaaggccggtatcgcaactaaccccaaagagccctgtgtgtgcacctgtcataacaaccctggagttaaagcaaactgctgccctgcacagctcggacttgctcagattagtgtaactgtgatgaaggcaacaggtctgtggggagattacttctcacagactgatggctatgtcaaggtcctgagaaatcataagttctttctaggggaaacatcagtgatttggaaccaaaactcaccaacctggaactggaattttgatctcggcagtttcgttctctctcagtttggggggctgagactagaagtgtgggatagggacaacaagtgggatgatgacctcctgggggcgtgcaacatacaactaaaagccggggtaaaaagcgacttctgtcgacttaaccatggtttgctgtactacaaaacgaatgtaacttgtgctcctagtttagctggcccctcatgcacccagtacgtgggctctccgatggcctcccatctagagaaggtatatgtgtcacgacacgcccgtcccatccccaaagatgagctggtgaaaatgggggtgcttttagatgagcgccgctttttgttaagtcaaaccagtgatcctaaaagcaaaacgcagactttgtgaaagcttatcatcaaagcacaataaaataagt

Intron 1

gtaagtgaaaaagagcttaaactcacttaattgtgagtttattgactgataataatacctatattaatatcttcaagctattttttaactactttaaactcctgtaaaacaaataaataaaataaaataaatgatgatgctgaagcactaacagcgttaaaattattctagcgattttgcatgggaacattataaatttaaactgctaaaatctagcatgcctatagaatgattcattctaaaaacattaaaacttataaggaagggtctaggatacaccaatcaaatcctgcacaaaggttttgattagaaccaatatatgagcaacaaagtttatatattaacatttgcactgaggttaacagtattaatattgtgctcttataatcagtaattttttttttttggtcttatttctcatttatgtatacgtatctcagcag

Intron 2

gtaagacttaacatatattagttaacttctgtgatgaatatcatggtatatgggttttaatgtgcagcattataaactgctgtcttcag

Intron 3

gtaagccaaaatatatcagtatcaatgtgtgctatttctctcagtctattgcatatatgtcactgttgcttgtaatctgttag

Intron 4

gtttgtgctttgaattggcagaatgtgatttaattgaattatcagctaaattattagttaaaaatggctaaacaatatagtctgatataaatattagttacataaatatagttttgtttgtgtgtgtgtatagtttataatattttttatttcatatttttaaatgtatattttaatttataatatttgtaatataattaatacatttttatttatggtttattttttgtattacttttttattatattatgtaattaaaattatacttataataataagaataatattttaatatttaataaatacatttaattggggg

aatctaaattttaataaattaaatttaataaatgttaaaaatttaatattttccttgaaaggtagtacattacgatacgatagatagatagatagatagatagatagatagatagatagatagatagatagatagatagatagatagatagatagatagatagatagctatagggttcaagcttaattttctttctttcttttctacaag
