# Supplementary material for: Molecular characterization of three novel perforins in common carp (Cyprinus carpio L.) and their expression patterns during larvae ontogeny and in response to immune challenges
Source: BMC Vet Res. 2018 Oct 3;14:299. doi: 10.1186/s12917-018-1613-y (PMC6169072; doi:10.1186/s12917-018-1613-y)
Supplement: Supplementary file 7 — Intron and exon sequences of CcPRF2. (DOCX 14 kb) [file 12917_2018_1613_MOESM7_ESM.docx]

Extron 1

aagactgttaagaaacgtctcaactag

Extron 2

cttgcctttgcaaatggctcccctgctgttgttcctctatcttcccattgccttctgttgcgaaatggcccccagcacagagtgcgaaaagcttcccttcgtaccgggacataatttggtgggagaaggctttgacattgtgcaaatgaagaccaccggagcttttgttgtggatgtgcggacctacatgaccggaggagagcatggtaactgcactcaatgtgagaacaaactgctcaacgagaagcagaagttgccagcttctgtggtggactggcgggtcaaggtgcagtgtcgccgcagtgttggcgccaagatttacgaatcggccagctcggttcttaaggacaccaccaattcagccagtgccagctggaagatcggactgagtgtgccgatggtgggaggtgtggctgttggtggcactcattcacgttcagccaggtttgccaaaagccacgcagctcaagacaagttctccttcacaagccacaccttctcctgtcgctactactc

Extron 3

atttcgtcttcatgctcgccctccactgaccaaagaatttttggggtccatcagttcacttcctgctaaatacgacagcaagtcagaaggtgcgtacaatcatttcatctcaatctacgggacacacttcttgcggagagtcgacctgggcggccgcgtgcattcaaccactgctgtgaagacctgccaggtttccatgaagggtctgtcagtacatgacgtgagcaactgcttatcggcggaggcgtccgccgtcatcaagggcgtgaag

Extron 4

gtgagtgggcaagcaggcttctgcaaaggcaaacaacagaaactggagaaaggcatcagcttcagcgcctccttctcagaccgagtcactgaaatactagggggcaacggtcagcagcaagacatcctcttcaatccaaacaacgttagtggttatgacacgtggctcaagtcactgaagaaaatccctggagtggtttcctacaccttgagctcattgcacatgctgttaaatcacgacccagctagacgagccagtctacaagcggccatcagcaagtacatcacaaaaagcgccatatctactgcttgcccctccaaatgcaaagtgggccatcgtagcaatagctgcacatgcaaatgcatcggtcacaaaagtattgatagaaactgctgcccaagtaatccaggagtggccacattgaccgtgactgtgttgagaggtgcaggattatggggagactatttctctaaaacggatggttatgttaaagtgttttacggaagccgtgcatatgagacccctgtgatttggaacaacaattttcctcagtggaactacaacataaatttggcaactgtagatctgtataaaaaaac

Extron 5

gacgttggtatttgaggtgtgggaccgtgacaaccggtttgatgatgatctcttgggaaagggatctcttgttccaaaaaaaggaaccaatcagaggcggtcatttaagttaaagcatggctcttttatgatttcctactctgcacagtgtggcccaagtcttaccgggtctttctgtgagaaatatgctcctacgccgggtggtgacggtactctaaactattaccagccctatggcgaagaacagcccattttgtttaaaaaatctgagggttttagaagaaatgtgtctttgctgaaataatacaacaactgcactgaatgtgaagataattgacttatttcttgacaaatttacaagtcttatttcaatagacacataaaactttatctgtttttttttaacattttttaaataatttcctctgattttaaaaaaaaaatattcttaatttgtttcataattccatatgtatactataaccaccatattgagatttttaaaaaaaatgaatgctttcagatgcaataaaaactttcttg

Intron 1

gtaaatatcagcatgcatggtttattatttggttcattattcagtagtcattttgtgaacggttcacgattaactgactgtaaatagaatgcatgaatattgatcctcatctttttaaaaactatgttttagttggcagattttatgatgctttgagtagctgcaccttattcatgtattcgatgtgacagtgtcactactctgtgttatttgacttaacgttgagcactttaattttaacttagattttaatcttaaattgaaagtaatttaattgaattgaaaacatgtgaatgtgttttttgggagtggatgtctatttatttagcaggtattttgcatcatggtttggacacactttaactgaatatatgtttctcttaatttttttaaaccttttaatccacagcctcccacttatatgtttgaaattcatcttgtaaactgctaaatataaagataatatttgcaacctgcatccttctgaagttgatttcatgattatattcttttggatggcatgtaaaaacagagaaaattttacatttaataactgtctatgcaagcagagcaccagagttggtatttgacatatttattgtttaattatctgtgaagcccgtgaagagctgactgtttttcggtgtataggacagtgatggaaactgcaataatgtagaagtaatgtgtgttgcactcaaaatatctagtggctttaatgcggcatcattttttagctgaaaaagtcacagttatccatcaactggcaaaatattagtatgcttcatactcaaatgtatgtgtagatactgtaaaacacttaagtcagctaaaattatatagaaaattcaaataacttcatttgatctatttgatatatcagcattcataaaggagggttgaatttctgattaagtcattttagagagaaatagaagatgttctcagctttttttcttttttttctacatttggaaatttggagattttgttatataatgcgaagatattcagactttttacatgcaaagtgtctatatagttcttggggctgtcgttttgtccaaatctaagattgtttattaattcagctttataattaaaattcctttttctctctctttcag

Intron 2

gtaagttctggtatatatgtgtgtatgtgtgattttttttttttttccacttgctattgtttatttgtaactacattcttaaatataaaggttatttatggttccatgaagaacctttaacatcaatagaagcttttcattccaaaaaaaggttctttacactcttaaaaataaagcatccaaaagggtttttcaattccatagaaccattttgggttcccaaaaggaccttttagttaatagttcttataagagagttttttcttaatgtgaagaacatttcaatattctaaagaagctttatccactgcaatggaccttttgtgcaatggacaggttgcatagatgttaaagattcttcatgggcccatcaatgccaataaagaacctttatttttaagtgtgagaagtggaaaaaggttaaatagtaattggtttaaaaatgtaactggctactctatcattggttatttgttgctaaacaaagtaaaattctagaatcgcatgctaaacagcgctcagctcattaaatattcatgagctcggtacagtcatagaagttgcgtacgcaattttccacgcacatatcaggatttataaaaaataaacttggcttaaatatgtatgcaccttacggacattctagaccatgcgtacacactcttcttcctggcgagaaaagtaatgaagtaagcaatgattttaaaatctgttttcttttctatatacacatttacattaaatattccactctcattaatggagataagcactgaaaaaacaaagtcattacgcagaatttaaacaaaaatgatatgtatttagacatatttatggaatgctgttttaatgacagcgatgagtgctataggtgcacactaattaatctttaaacttaactgcagatctaatgctatgcataaatattttaaggagaacaatgatcaatgaggcgcacttacttcgatattattgcacacactgctggattcagtgatcgaacggtctgttgtttgaataggtccaatgataatatcttactgtacaaccacagctgcacggaggtgttgatgccatgtgaaggcatctccacaacattatgaaaaataccactgtatttgaaggataaaacttgaagaaaatggtaatttgcatgtttctttttaaaataggctactttatcagtgacgtgccgagtcagacaattctatttacactgcaataaatataggctaaacatagctaaaagatgttcaccttatcagcaaaactgcataaattaaatttgattttaattgtttgaaacaactgaaatactatttggccagtggaaaagaatgagaccaactctattctaaaataatcatctgagaactattttaaacagttttgtttttatggatttttatttttatatatttatgctaaaacataacaaggatactggatgatttttagctataaaaattgtgtatggcacaaatggcatttatagtccatatcttatatttcctataatgtcttgtacctttgtctgtgttaaacatggtgtcacgtgcatgggaatatgcatggaaataatatgcagatgaggttatgcatagtaaaactaggtgttgtaagctccatatatagtgatttcggggaggagtaggtgtggagatgcacgtatgcccaatcttcccctgactgggatttataaagggatttttgagcaggttctggcgtacacatggttttatatatctgaaaacttttgtgcgtacgcaaaatctagcttttgtgcatacgtacacttttaggatgaaatctacagagagttttataaatgagacccctagaccttagtcagggtagcaccatattccatttttgtcaggagtggaaacgaggctgtaatatgaggaacagtgtgcataatggattgtactgttgtttaatgttgtctttcagtatagacaaaaactccatgaaacagttttgaaagttgtgacttgtaaaaattatgtgatctacactcagaaaaaaggtacaaaagcacactggtgtggtaccttttcaaaatgtacacttttgtgccctttaggtgctaatatgtacttatatggaccatttagatacaaagatgaatcttttgaaaagctatcgtcccagtaacagcttttgtacctttgtgtagagctttatacagtgcgtccaattgtaaatactataagtctatccctcacagccttgttttaatttgccaaaaaaaacagtatgtcatctaatctgactataaatgtctataaagtcacaattacccaattttttatgtttttattttttattgttttactctacggcagaatcaggcttccatattgaacaaagtgaaatgtgttacaagatcacccaggattacattagtctggggtttggactgacaatgaagcatgaaaaccctataaagtctttcttctcttctttcatcag

Intron 3

gtgagtgggcaagcaggcttctgcaaaggcaaacaacagaaactggagaaaggcatcagcttcagcgcctccttctcagaccgagtcactgaaatactagggggcaacggtcagcagcaagacatcctcttcaatccaaacaacgttagtggttatgacacgtggctcaagtcactgaataaaatccctggagtggtttcctacaccttgagctcattgcacatgctgttaaatcacgacccagctagacgagccagtctacaagcggccatcagcaagtacatcacaaaaagcgccatatctactgcttgcccctccaaatgcaaagtgagtacatgacgtgagcaactgcttatcggcggaggcgtccgccgtcatcaagggcgtgaag

Intron 4

gtaagtggttacattcacaagacttaatcttagtttcagtttcggtcattgggtgatacctgttgtacttcatatcttgtctatgctcattttacag
