# Supplementary material for: Molecular characterization of three novel perforins in common carp (Cyprinus carpio L.) and their expression patterns during larvae ontogeny and in response to immune challenges
Source: BMC Vet Res. 2018 Oct 3;14:299. doi: 10.1186/s12917-018-1613-y (PMC6169072; doi:10.1186/s12917-018-1613-y)
Supplement: Supplementary file 8 — Intron and exon sequences of CcPRF3. (DOCX 13 kb) [file 12917_2018_1613_MOESM8_ESM.docx]

Extron 1

atcatatgaaatgagaagttagagagagaacatatttcattttgatctgttcacctataaattcaaatg

Extron 2

atcatggggaagttccagatttacctaattttcttggcattgcttctcatgaccatttctggagaagatgacttaggtgcatcaaaagagtgtaaggatgctccctttgttccaggatacaatcttgctggagaaggatttgatgttgtgaccatggagcgaaaag

Extron 3

gttcttatgtgatcaacacagaaatatgggatttaggaaatggcacttgtaaattacgcaaaaacaagtacatgaatggaataaaacagaagctaccagcagcagttgtagactgggggaccttgccgaagtgttcaatgaaagtctccagtcagatctttgaatcagctgaagcactagtcaatgattcattatcagcactcccagatggttggaaagtaggtttagatgtgaaggctgttggagctgcaattggaagcactcattccagggaagcaaaatttgcaatgacaaaatcaaaagaagacaaatacagtttcaccaaacatgaagcagggtgcaatatttacag

Extron 4

atatcgtatatctgaaaagcctcctcttcatgaagagtttcttgaagcaatcaaatcactccctgcatcctacgatccagatgcctaccgcaacctgatctccacatatggcactcactataccacaagtgttaagttagggggtcaaatgaaagccataaccgccatcaaaacctgtcaggcagcagtgagtggacttacagacactgcggtaaaggactgtttggatgtggaagcttctggttcacagagcacagcgactgtgaacgaaaagacacagttttgtcgagaacagaaaaaaaagatgcacacaaatgaaaagttcagctccatgttcagtgagcgtcagacagagattattggaggaaacataaatggagaagatctgcttttttctggttcatcacacccagattcccttaaaaaatggcttgagtctttgaagagtctccctgacattgtgcattactctctgaaacccctccatttcttactgagtaataaacaccctgcaagaaaaggactgaagaaagctgtagaagaatacatcatccaaaatgccctcatggagatttgctctgagccttgcaagattggcaggaagtgcagtgcaagagaccgatgtgcttgtgtttgtgaaagtagtcaatttataaagtctaactgctgtccagctgaaaaaggacttgccaccctgaaagtctacaatctcagagccaaaggtctgtatggagatgtaggtagtcaaacagatggaactgtaactgttacatatgacaaacaaatcaagcgcactgagaccattgataataatgacaatccacgttggccagaaacatttgagtttggacctattaagatcagaaagtccagtaacctaacttttgaagtatatgatgcagacagctattggaacagcgatcttcttggtaaatgctctttttatctaagaagtggagttgtggatgatgcttgtgtttttacatatggcaccttctattttacctatgaagtgaaatgtgcacccagtctgcagggcccacagtgtaatgaacacaaaccttcaccaatggctgcccatctggctgatattttcagctcaagaaatggtgttctggttaaagacctgccgaggcttaaactagctctcaattactcagataagttcaatttcaagagcacctatggaaagctgatgatgctgtgacctgcattttgaatccctcctttatgcatgaaaaatatccctaatatggcccaacagaactttcatttgctagaaaaaagagtatatctgcaacatttgcttacttgtattcccatttcctttctgcgttcttgcattaataaagcattcacaaagacag

Intron 1

gtaagaaactataaaaaatcatcaaattaatcatatgattttaaagaaaccattagtgttttacagtacatacttgtacatactatttgtttttaaaataatattgttgatataataaattttgtgaagaagtttattgttatgtaaaatctgacaccattaatgacttatctactttcactacagatcatggggaagttccagatttacctaattttcttggcattgcttctcatgaccatttctggagaagatgacttaggtgcatcaaaagagtgtaaggatgctccctttgttccaggatacaatcttgctggagaaggatttggatgttgtgaccatggagcgaaaaggttcttatgtgatcaacacagaaatatgggatttaggaaatctgtactacctaataatacaaatggtaanaactaataaaaatacatcaaattaatcatatgtattttaaagaaccattagtgttttacagtacatacttgtacatactatttgtttttaaataatattgttgatataataaattttgtgaagaagtttattgttatgtaaaatctgacaccattaatgacttatctactttcactacag

Intron 2

gttcttatgtgatcaacacagaaatatgggatttgatacaatcttgctggagaaggatttgatgttgtgaccatggagcgaaaag

Intron 3

gttatttttcctctctcttactgttaaaaagtacaatgactatgaaattaaaactttaattgcaaattgttccttctttgattccactaagtttaaatgagtttattgtttctttatag
