# Supplementary figures and images for: Molecular characterization of three novel perforins in common carp (Cyprinus carpio L.) and their expression patterns during larvae ontogeny and in response to immune challenges
Source: BMC Vet Res. 2018 Oct 3;14:299. doi: 10.1186/s12917-018-1613-y (PMC6169072; doi:10.1186/s12917-018-1613-y)

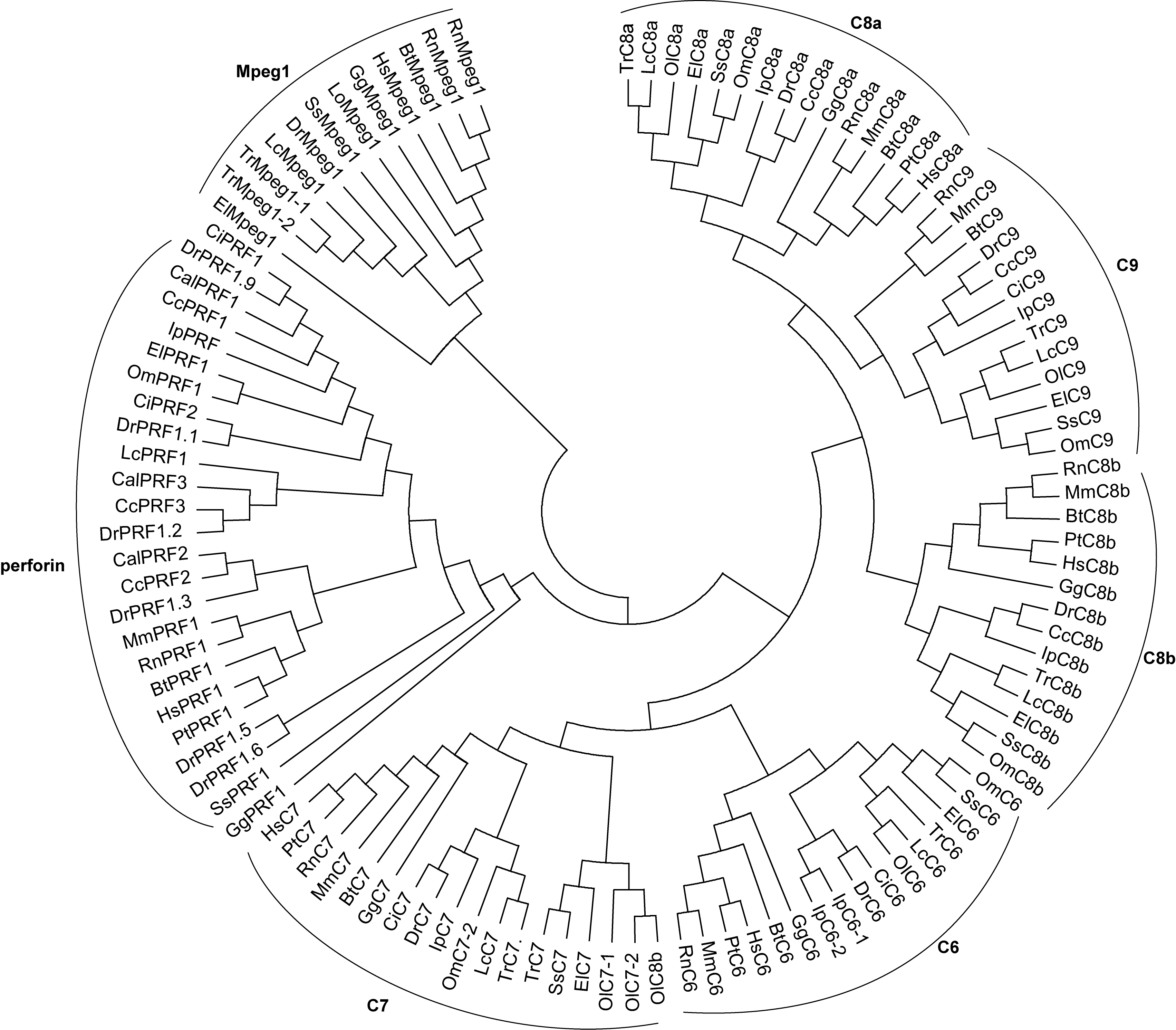

Supplement: Supplementary file 9 — Figure S1. Phylogenetic analysis of perforin, Mpeg-1, C6, C7, C8 and C9 polypeptides. This tree is generated using the neighbor-joining (NJ) method in MEGA 6.0. GenBank accession numbers used are shown in Additional file 4: Tables S4 and Additional file 5: Table S5, and the species abbreviations in Fig. 3. (TIF 29791 kb) [file 12917_2018_1613_MOESM9_ESM.tif]
