# Supplementary material for: Molecular characterization of three novel perforins in common carp (Cyprinus carpio L.) and their expression patterns during larvae ontogeny and in response to immune challenges
Source: BMC Vet Res. 2018 Oct 3;14:299. doi: 10.1186/s12917-018-1613-y (PMC6169072; doi:10.1186/s12917-018-1613-y)
Supplement: Supplementary file 10 — ARRIVE (Animal Research: Reporting of in Vivo Experiments) checklist. [file 12917_2018_1613_MOESM10_ESM.docx]

Thank you for the final proofs. We have reviewed the manuscript and supplemental files and the only revision required is to Table 2, where it appears that footnote c is missing. It should read "^c^ These studies were relevant to child health but included adults only (e.g., outcomes in adults related to an exposure in childhood; intended to include both children and adults but no studies with children were found)". No other changes are required. Thanks,

Michelle
